# Gut microbiota-mediated betaine regulates skeletal muscle fiber type transition by affecting m^6^A RNA methylation and *Myh7* expression

**DOI:** 10.1080/19490976.2025.2545434

**Published:** 2025-08-18

**Authors:** Chao Yan, Yilong Yao, Zhaobo Zhang, Fanqinyu Li, Danyang Fan, Wen Liu, Xinhao Fan, Lingna Xu, Yanwen Liu, Shilong Wang, Mengling Hu, Yalan Yang, Zhonglin Tang

**Affiliations:** a Shenzhen Branch, Guangdong Laboratory of Lingnan Modern Agriculture, Key Laboratory of Livestock and Poultry Multi-Omics of MARA, Agricultural Genomics Institute at Shenzhen, Chinese Academy of Agricultural Sciences, Shenzhen, China; b Kunpeng Institute of Modern Agriculture at Foshan, Agricultural Genomics Institute, Chinese Academy of Agricultural Sciences, Foshan, China; c Key Laboratory of Agricultural Animal Genetics, Breeding and Reproduction of Ministry of Education & Key Lab of Swine Genetics and Breeding of Ministry of Agriculture and Rural Affairs, Huazhong Agricultural University, Wuhan, China; d Department of Molecular Biology and Biophysics, University of Connecticut Health Center, Farmington, CT, USA

**Keywords:** Betaine, gut microbiota, myofiber-type transition, *Myh7*, m^6^A RNA methylation

## Abstract

Skeletal muscle fiber composition is essential for maintaining muscle function and overall health. Growing evidence underscores the pivotal role of the gut-muscle axis in mediating the influence of gut microbiota on skeletal muscle development. However, the mechanisms underlying microbiota-mediated regulation of skeletal muscle fiber type remain unclear. Here, we employed multi-omics approaches, including RNA-seq, MeRIP-seq, 16S rRNA gene sequencing, and metabolomics, to investigate the causal relationship between the gut microbiota and skeletal muscle fiber transition. Our results demonstrate that the gut microbiota modulates skeletal muscle fiber transition by influencing N6-methyladenosine (m^6^A) methylation to regulate the expression of the slow-twitch fiber marker *Myh7*. Specifically, METTL3-dependent m^6^A methylation enhances *Myh7* gene expression, leading to an increased proportion of slow-twitch fibers and a reduction in fast-twitch fibers. Furthermore, the microbiota-derived methyl donor betaine promotes *Myh7* expression and *Akkermansia muciniphila* (*AKK*) abundance, and facilitates fast-to-slow fiber conversion via m^6^A modification. The transplantation of *AKK* significantly altered betaine levels and m^6^A modification, thereby promoting muscle fiber remodeling. In conclusion, these findings reveal that *AKK*-coordinated betaine drives skeletal muscle fiber conversion by modulating *Myh7* mRNA expression. This study provides novel insights into the role of m^6^A RNA methylation in the gut-muscle crosstalk, highlighting potential therapeutic targets for muscle-related disorders.

## Introduction

Skeletal muscle comprises a heterogeneous population of fibers with distinct contractile and metabolic properties.^1^ Based on contraction speed and metabolic profile, muscle fibers are broadly classified into two main types: slow-twitch (type I, primarily expressing myosin heavy chain 7, *Myh7*) and fast-twitch (type II, including subtypes IIa, IIx, and IIb, predominantly expressing *Myh1*, *Myh2*, and *Myh4*, respectively).^2,3^ Notably, type IIb fibers are present in rodents but absent in humans.^4^ Slow-twitch fibers are distinguished from fast-twitch fibers by their higher mitochondrial density, greater oxidative capacity, and increased myoglobin levels.^2^ Importantly, muscle fiber composition is highly plastic, allowing adaptive transition in response to physiological or environmental stimuli.^5,6^ A shift from fast- to slow-twitch fibers enhances fatigue resistance and sustains long-term energy supply.^7^ Thus, understanding the mechanisms governing fiber-type transition is essential for optimizing muscle function and systemic health.

The gut-muscle axis implicates gut microbiota as a critical regulator of skeletal muscle physiology and metabolism,^8,9^ influencing muscle mass,^10^ fiber composition,^11^ and functional performance.^10,12^ Nevertheless, there is a significant knowledge gap regarding the precise roles of gut microbiota in governing muscle phenotypes. Bioactive metabolites derived from the gut microbiota, such as short-chain fatty acids (SCFAs),^13^ have been shown to modulate muscle satellite cell homeostasis,^12^ attenuate muscle atrophy, and influence neuromuscular junctions in mice.^10^ Preliminary evidence from pigs and mice suggests that microbiota may regulate muscle fiber-type composition by altering the expression of slow-twitch fiber marker *Myh7* and fast-twitch fiber marker *Myh4*,^11^ yet the underlying mechanisms remain elusive. We therefore postulated that microbiota-dependent metabolites play a regulatory role in muscle fiber transition.

Betaine (*N*-trimethylglycine), a natural substance found in food, is metabolized by the gut microbiota, which in turn shapes microbial ecology.^14,15^ Betaine exhibits osmoprotective functions, supports glucose homeostasis regulation, and has been associated with anti-obesity properties.^16^ Notably, betaine has been shown to directly modulate skeletal muscle fiber-type composition.^17^ In mice, exogenous betaine supplementation ameliorates high-fat diet-induced gut microbiota dysbiosis and enriches beneficial microbial strains.^18^ As a methyl donor in the methionine cycle, betaine enhances hepatic mRNA methylation.^19,20^ However, whether microbiota-derived betaine regulates skeletal muscle fiber profiles via DNA/RNA methylation remains unknown.

N6-methyladenosine (m^6^A) dynamically regulates gene expression through RNA stability, translation, and splicing.^21,22^ It plays a critical role in skeletal muscle growth and maintenance. Emerging evidence reveals that gut microbiota substantially influences m^6^A RNA methylation patterns across multiple tissues, including the cecum, brain, intestine, and liver.^23,24^ Specific microbial species, such as *Akkermansia muciniphila* (*AKK*) and *Lactobacillus plantarum*, can affect m^6^A RNA modifications in murine cecum and liver.^23^ Of particular relevance to muscle biology is the enzyme methyltransferase-like 3 (METTL3), a key methyltransferase responsible for m^6^A modification, which functions as a critical regulator in muscle maintenance and growth in mice.^25–27^ Based on these observations, we hypothesized that gut microbiota-mediated betaine may regulate muscle fiber conversion via m^6^A RNA methylation.

The study aimed to elucidate how gut microbiota-mediated betaine modulates skeletal muscle fiber composition through m^6^A RNA methylation (Supplementary Figure S1). Leveraging multi-omics data, including transcriptomics, microbiomics, epitranscriptomics, and metabolomics, we identified that METTL3-dependent m^6^A modification acts as a mechanistic link between the gut microbiota and muscle fiber remodeling. Through complementary *in vitro* and *in vivo* validation, we discovered key microbial taxa and microbiota-derived betaine as regulators of muscle fiber transition, offering new insights into the gut-muscle axis. Our animal-focused findings establish that the “gut microbiota-betaine-m^6^A-*Myh7*” axis governs muscle plasticity through post-transcriptional regulation. These findings advance understanding of the molecular basis of muscle plasticity and highlight promising therapeutic strategies for modulating skeletal muscle in both clinical and agricultural applications.

## Materials and methods

### Ethical approval and animal husbandry

All experimental protocols and animal care procedures were conducted in strict accordance with the guidelines established by the Chinese Academy of Agricultural Sciences and the Institutional Animal Care and Use Committee (approval No. AGIS‐ER‐2024‐006). The study followed the ARRIVE 2.0 guidelines for reporting animal experiments.^28^ Female C57BL/6J mice (8–10 weeks of age), obtained from GemPharmatech Co., Ltd. (China), were maintained under either germ-free (GF; *n* = 12) or specific pathogen-free (SPF; *n* = 20) conditions. Conventional (COV; *n* = 12) C57BL/6J female mice were housed under standard housing conditions with a 12-h light/dark cycle, an ambient temperature of 24–26°C, and a relative humidity of 40–60%. All animals had *ad libitum* access to food and water until euthanasia by CO₂ inhalation followed by cervical dislocation to ensure death. Mice were fed a standardized diet (1010013, Xietong Shengwu, China), which was completely sterilized by irradiation. To minimize cage effects, mice from at least three different cages were included in each experimental group. Animal health and well-being were routinely monitored throughout the study.

### Antibiotic cocktail (ABX) treated mice

Female C57BL/6J mice (6 weeks of age) were administered a broad-spectrum antibiotic cocktail via the drinking water from 6 to 12 weeks of age to deplete the gut microbiota, following a previously established protocol.^10^ Mice were randomly assigned to two groups: an ABX-treated group and an untreated control group (*n* = 10 per group), under standard raising conditions. The antibiotic cocktail consisted of ampicillin (1 g/L; AA022, Genview, China), neomycin (1 g/L; AN214, Genview, China), metronidazole (1 g/L; Cat. #HY-B0318, MCE, USA), and vancomycin (0.5 g/L; Cat. #HY-B0617, MCE, USA). For downstream analysis, mice were euthanized using CO₂ inhalation, and gastrocnemius (GAS) muscle and fecal samples were rapidly collected under sterile conditions, snap-frozen in dry ice, and stored at −80°C until further processing.

### Gram-staining

To visualize bacteria, Gram-staining was performed on fecal contents using a commercial kit (R40080, Thermo Scientific™, USA), according to the manufacturer’s instructions.

### Colon microbiota analyses by 16S rRNA gene sequencing

Genomic DNA was extracted from the samples using the cetyltrimethylammonium bromide (CTAB)/sodium dodecyl sulfate (SDS) method. DNA concentration and purity were assessed by 1% agarose gel electrophoresis, followed by spectrophotometric quantification using the A260/A280 ratio. The V3-V4 hypervariable regions of the 16S rRNA gene were amplified using the primer pair 341F-806 R (forward: CCTAYGGGRBGCASCAG; reverse: GGACTACNNGGGTATCTAAT). Each sample was labeled with a unique dual-index barcode during PCR amplification. Sequencing libraries were constructed using the NEB Next® Ultra™ DNA Library Prep Kit (NEB, USA), following the manufacturer’s protocol, including adapter ligation and size selection. Paired-end sequencing was carried out on the Illumina MiSeq/HiSeq2500 platform.

Raw paired-end reads were merged using USEARCH (v11), and the merged sequences were subsequently quality-filtered, length-trimmed, and dereplicated to obtain unique sequences. Sequences with ≥ 97% similarity were clustered into operational taxonomic units (OTUs). Taxonomic classification of OTUs was performed by aligning representative sequences against the ribosomal database project (RDP) 16S database (rdp_16s_v18.fa). Alpha-diversity analysis and differences in microbial community composition between groups were evaluated using analysis of similarities (ANOSIM) based on Bray-Curtis dissimilarity matrices via the vegan package (v2.6–4) in R (v4.4). The relative abundances of bacterial phyla and genera were visualized using the circlize (v0.4.15) and ggplot2 (v3.4.4) packages in R (v4.4). To predict the functional potential of the microbial communities, PICRUSt2 (Phylogenetic Investigation of Communities by Reconstruction of Unobserved States) analysis was performed using the PICRUSt2 software (v2.6.2).

### Quantitative detection of RNA m^6^A level

The m^6^A methylation status of purified mRNA from GAS muscle was assessed and quantified using the EpiQuik™ m^6^A RNA Methylation Quantification Kit (#P-9005, Epigentek, New York, USA) by enzyme-linked immunosorbent assay (ELISA), according to the provided instructional protocol.

### Methylated RNA immunoprecipitation assay and sequencing (MeRIP-seq)

To delineate the landscape of m^6^A modification in skeletal muscle under varying gut microbial conditions, we performed a MeRIP-seq analysis comparing GF, SPF, and COV mice. Briefly, polyadenylated RNAs were extracted from skeletal muscle and fragmented to 100–200 nt. These RNA fragments were then incubated with or without anti-m^6^A antibody (Synaptic Systems 202,203). m^6^A-modified mRNA fragments were selectively captured using Dynabeads Protein A-bound antibody complexes. The bound RNAs were washed and eluted through competitive binding with free m^6^A nucleotides. Library construction included reverse transcription, second-strand cDNA synthesis, end repair, 3′-adenylation, and fragment selection. Sequencing was performed on the Illumina HiSeq platform (Seqhealth Technology Co., LTD, Wuhan, China), with three biological replicates per group.

Clean reads were mapped to the mouse reference genome (mm10) using HISTA2 (v2.2.1) and processed with SAMtools (v1.22) to generate indexed BAM files. Peak annotation was performed using ChIPseeker (v4.5), and signal profiling and heatmap generation were conducted using deepTools (v3.5.5). Different m^6^A peaks were identified using exomePeak2 (v1.14.3) (|log_2_ Fold Change [FC]| > 1 and *p* < 0.05). Motif enrichment analysis was conducted using the findMotifsGenome.pl function of HOMER (v4.4). Genes adjacent to different peaks were enriched for Gene Ontology (GO) enrichment analysis for biological process using the clusterProfiler package (v4.10.0). Peak visualization was carried out using Integrative Genomics Viewer (IGV, v2.16.0).

### Transcriptome sequencing (RNA-seq) analysis

Total RNA was extracted from skeletal muscle tissues of GF, SPF, and COV mice using TRIzol™ Reagent (Invitrogen, Cat. No. 15596026, USA), following the manufacturer’s protocols. RNA concentration was measured using a Qubit™ 3.0 fluorometer with a Qubit™ RNA Broad Range Assay Kit (Life Technologies, Q10210, USA). For RNA sequencing, 2 μg of polyadenylated RNA per sample was used to construct stranded RNA-seq libraries following the manufacturer’s guidelines. High-throughput sequencing was conducted by Seqhealth Technology Co., Ltd. (Wuhan, China).

Raw sequencing reads were quality-filtered to obtain clean reads, which were aligned to the mouse reference genome (mm10) using HISAT2 (v2.2.1), and then processed with SAMtools (v1.22). Gene expression levels were calculated as transcripts per kilobase million (TPM) using StringTie (v1.3.1), with the GENCODE M25 annotation as the reference. Principal component analysis (PCA) was performed to assess sample clustering and intergroup variation. Differential gene expression analysis was conducted using DESeq2 (v1.42.0), with the criteria for differentially expressed genes (DEGs) defined as |log_2_ FC| ≥ 1 and false discovery rate (FDR) ≤ 0.05. GO and Kyoto Encyclopedia of Genes and Genomes (KEGG) enrichment analysis were performed using the clusterProfiler package (v4.10.0) in R (v4.4). Weighted gene co-expression network analysis (WGCNA) was conducted using the WGCNA package (v1.72)^29^ to identify gene modules and associated biological pathways.

### Single-gene gene set enrichment analysis (GSEA)

In the context of GSEA, no arbitrary significance thresholds were applied when interpreting gene expression data.^30^ Specifically, for correlation-based single-gene GSEA, the expression of the target gene (*Mettl3* or *Ythdf2*) was used to calculate Pearson correlation coefficients with all other genes across samples.^31^ The resulting pre-ranked gene lists were then analyzed using the clusterProfiler package (v4.10.0)^32^ in R (v4.4). This approach enabled pathway-level interpretation of gene expression patterns associated with *Mettl3* or *Ythdf2* expression, providing insights into the underlying molecular functions.

### Cell culture and transfection

The mouse myoblast cell line C2C12 and human embryonic kidney 293T cell line were obtained from the American Type Culture Collection (ATCC). All cell lines were regularly tested for mycoplasma contamination using a PCR-based Mycoplasma PCR Detection Kit (C0301S, Beyotime, China), and only mycoplasma-negative cells were used for experiments. The growth medium consisted of 90% Dulbecco’s Modified Eagle’s Medium (DMEM; Gibco, California, USA), supplemented with 10% fetal bovine serum (FBS; Gibco, California, USA), and 1% penicillin–streptomycin solution (PS; Thermo Scientific, Massachusetts, USA). During myogenic differentiation, C2C12 cells were transferred to a differentiation medium containing 97% DMEM, 2% horse serum (Bioind, Shanghai, China) and 1% PS. All cells were cultured in a humidified incubator at 37°C with 5% CO₂. Transfection was performed using Lipofectamine™ 3000 (Thermo Fisher Scientific, USA), following the manufacturer’s protocol.

### Plasmid and oligonucleotides

Custom-designed small interfering RNAs (siRNAs) targeting METTL3 (si-Mettl3) and YTHDF2 (si-Ythdf2), along with a negative control siRNA (si-NC), were synthesized by RiboBio (Guangzhou, China), and were provided in Supplementary Table 1–1. For gene overexpression, pcDNA3.1-Mettl3 and pcDNA3.1-Ythdf2 expression plasmids were constructed and synthesized by GeneCreate (Wuhan, China), with the plasmid details listed in Supplementary Table 1–2. Additionally, for the dual-luciferase reporter assay, a DNA fragment of the *Myh7* gene containing m^6^A binding sites was cloned into the pGL3-Basic vector.

### Immunofluorescence assay

C2C12 cells were seeded into 12-well plates and cultured in differentiation medium for 48 h post-transfection. GAS muscles were embedded in optimal cutting temperature (OCT) compound, snap-frozen in liquid nitrogen, and cryosectioned into 10 µm-thick slices. Both cells and tissue sections were fixed with 4% paraformaldehyde for 15 min (cells) or 30 min (tissue sections), washed with phosphate buffered saline (PBS), and permeabilized with 0.3% Triton X-100 for 10 min. After permeabilization, samples were blocked in 5% bovine serum albumin (BSA) for 1 h at room temperature. For immunofluorescence staining, samples were incubated with primary antibodies against MYH7 (1:500, #64038, CST, USA) and MYH4 (1:500, MABT847, Merck, Germany) either overnight at 4°C or for 2 h at room temperature. Samples were then treated with an Alexa Fluor 488®-conjugated goat anti-mouse IgG secondary antibody (1:500, ZF-0512, Zhongshan Golden Bridge, China) for 1 h at room temperature in the dark. Cell nuclei were counterstained with DAPI (1:1000, C1002, Beyotime, China), and cell membranes were labeled with an anti-laminin antibody (1:1000, ab11575, Abcam, USA). Fluorescent signals were visualized using a fluorescence microscope.

### Real-time quantitative PCR (RT-qPCR)

Total RNA was extracted from cultured cells and skeletal muscle tissues using Trizol™ reagent (Invitrogen, Carlsbad, CA, USA) following the manufacturer’s protocol. Subsequently, cDNA was synthesized from high-quality RNA using the HiScript III 1st Strand cDNA Synthesis Kit (with gDNA wiper) (R323, Vazyme, China). For gene expression analysis, RT-qPCR was conducted using the ChamQ SYBR qPCR Master Mix (Q711, Vazyme, China). The target genes included *Myh7*, *Myh4*, *Mettl3*, *Ythdf1*, *Ythdf2*, and *Ythdf3*. *β-actin* served as the internal reference gene for normalization. Relative gene expression levels were calculated using the 2^−ΔΔCT^ method. Primer sequences are provided in Supplementary Table 1–3.

### Western blot

Proteins were extracted from C2C12 cells and skeletal muscle tissues using lysis buffer (78501, Thermo Scientific^TM^, USA). Western blot was performed according to a standard protocol, as previously described.^33,34^ Briefly, equal amounts of total protein were separated by sodium dodecyl sulfate polyacrylamide gel electrophoresis (SDS-PAGE) and transferred onto 0.22-µm polyvinylidene fluoride (PVDF) membranes. The membranes were blocked and then incubated with the following primary antibodies: anti-MYH7 (1:1000, #64038, CST, USA), anti-MYH4 (1:1000, MABT847, Merck, Germany), anti-METTL3 (1:1000, AV34590, Merck, Germany), anti-YTHDF2 (1:1000, #80014, CST), and anti-GAPDH (1:1000, #2118, CST, USA). Following primary antibody incubation, horseradish peroxidase (HRP)-conjugated secondary antibodies–goat anti-rabbit IgG H&L (ab6721) and goat anti-mouse IgG H&L (ab205719) (both from Abcam, USA), were applied for 2 h at room temperature. The membranes were then washed three times with Tris-buffered saline with Tween-20 (TBST) for 5 min each. Protein bands were visualized using a chemiluminescence imaging system (3500, Taenon, China), and band intensities were quantified using ImageJ software.

### Dual-luciferase reporter assay

293T cells were seeded into 24-well plates and grown until reaching 70–80% confluency. Cells were co-transfected with either the pcDNA3.1-Ythdf2 expression plasmid or the negative control plasmid (pcDNA3.1-NC), along with either a wild-type firefly luciferase reporter vector (pGL3-Myh7-WT, containing *Ythdf2* binding sites) or its mutant counterpart (pGL3-Myh7-MT). A constitutively expressed *Renilla* luciferase plasmid (pRL-TK) was also included for normalization. Transfection was performed using Lipofectamine™ 3000 (Thermo Fisher Scientific, USA) according to the manufacturer’s protocol. After 48 h, cells were harvested, and both firefly and *Renilla* luciferase activities were measured using a luminometer and the Promega dual-luciferase® reporter (DLR^TM^) assay system (E1910, Madison, USA). Relative luciferase activity was calculated as the ratio of firefly to *Renilla* luciferase signals.

### m^6^A dot blot analysis

Total RNA was extracted from *Ythdf2*-overexpressing myoblasts. Each RNA sample (200 ng/µL) was denatured at 65°C for 10 min, and immediately chilled on ice. The denatured RNA was then spotted onto nitrocellulose filter membrane (Millipore, Billerica, MA, USA). RNA was UV cross-linked to the membrane twice for 30 min each, and then incubated overnight at 4°C with an m^6^A-specific monoclonal antibody (1:1000, 68055–1-lg, Proteintech, USA). After primary antibody incubation, membranes were blocked with 5% non-fat milk in TBST for 1 h at room temperature, then incubated with an HRP-conjugated goat anti-mouse IgG secondary antibody (1:2000, ab205719, Abcam, USA) for 1 h at room temperature. The m^6^A signal was detected using a chemiluminescence imaging system.

### mRNA stability assay

C2C12 cells were transfected with either si-Ythdf2 or si-NC or with pcDNA3.1-Ythdf2 or pcDNA3.1-NC, as previously described.^35,36^ Following transfection, cells were treated with 5 µg/mL Actinomycin D (ActD; HY-17559, MCE, New Jersey, USA) to inhibit RNA transcription. After incubation for designated time points (0, 1, 3, 5, and 7 h post-ActD), total RNA was extracted and *Myh7* mRNA expression levels were quantified using RT-qPCR. The mRNA half-life was calculated by fitting decay curves to a first-order exponential decay model.

### Untargeted metabolome analyses of colonic contents, serum, and skeletal muscle

Colon content, serum, and skeletal muscle samples from mice (*n* = 6 per group) were rapidly frozen in liquid nitrogen immediately after dissection. The tissues were then transferred to Eppendorf tubes containing 200 μL of H₂O and ceramic beads for mechanical homogenization. Metabolites were extracted by adding 800 μL of a methanol/acetonitrile mixture (1:1, v/v), followed by centrifugation. The resulting supernatant was collected and dried using a vacuum centrifuge.

For liquid chromatograph-mass spectrometer (LC-MS) analysis, the dried extracts were reconstituted in a 1:1 (v/v) acetonitrile/water solvent. Metabolite separation was performed using a UHPLC system (1290 Infinity LC, Agilent) equipped with a HILIC column, operated in both positive and negative electrospray ionization (ESI) modes. Quality control (QC) samples were regularly interspersed throughout the analysis to monitor system stability and ensure data reliability. Mass spectrometry detection was conducted using an AB Sciex Triple TOF 6600 (AB SCIEX) under optimized ESI source conditions.

Raw MS data were converted to mzXML format using ProteoWizard MSConvert and processed with the XCMS package. Ion features were retained only for those with more than 50% nonzero values in at least one experimental group. Metabolite identification was achieved by matching the accurate m/z values (< 10 ppm) and MS/MS spectra to an in-house database constructed with authentic standards. Following sum-normalization, data were analyzed using the R package ropls (v1.34.0), including PCA. Metabolites with a variable importance in projection (VIP) score > 1 and *p* < 0.05 were considered statistically significant.

### Betaine treatment of C2C12 cells and conventional mice

*In vitro*, C2C12 cells were grown in 6-well plates and cultured in a growth medium (DMEM supplemented with 20% FBS) until reaching approximately 80% confluency. Then, the cells were switched to a differentiation medium (DMEM supplemented with 2% horse serum) and treated with graded concentrations of betaine (0, 5, 10, and 15 mM; Cat. No. 8230, Solarbio, China). Myotube formation was assessed by immunofluorescence staining for fast- and slow-twitch fibers.

*In vivo*, six-week-old female C57BL/6J mice were randomly divided into either a control or a betaine-treated group (*n* = 10 per group). Betaine was administered via drinking water at a concentration of 10 mM (as previously described)^17^ for a duration of 16 consecutive weeks. All mice were housed under standard controlled environmental conditions with free access to food and water. After the treatment, tissues and gut contents were collected for histological analysis or snap-frozen in liquid nitrogen and stored at −80°C.

### Redundancy analysis (RDA)

The relationship between gut microbiota and fecal metabolite profiles was investigated using RDA. This method was applied to identify specific microbial taxa associated with key metabolite variations, utilizing the RDA functions of the vegan package (v2.6–4) and visualization via ggplot2 (v3.4.4) in R (v4.4).

### AKK bacterial colonization of ABX pre-treated mice

*AKK* strain BNCC341917 was obtained from the BeNa Culture Collection (BNCC, Beijing, China). The bacterium was cultured in fluid thioglycolate medium (BNCC353538) at 37°C under anaerobic conditions using 2.5 L anaerobic sachets (Thermo Fisher, USA). For the animal model, female C57BL/6J mice (*n* = 12; 6 weeks old) were maintained under standard laboratory conditions. Prior to colonization, mice received an ABX pre-treatment for 5 consecutive days, following a previously established protocol.^37^ For fecal microbiota transplantation (FMT), ABX-treated mice were subjected to oral gavage with 10^^9^ colony-forming units (CFU) of *AKK* or 200 μL PBS per mouse. Fecal samples were collected 48 h after bacterial gavage to assess the colonization efficiency. The establishment of mono-colonization was monitored and validated through *AKK*-specific PCR, utilizing the forward primer 5’-CAGCACGTGAAGGTGGGGAC-3’ and the reverse primer 5’-CCTTGCGGTTGGCTTCAGAT-3.’

### Trimethylamine N-oxide (TMAO) targeted metabolome analyses of colonic contents

Metabolite analysis of colonic contents from mice treated with *AKK* or PBS (*n* = 4 per group) was performed following an established protocol. Briefly, 50 μL of colon content was mixed with 150 μL of methanol containing an internal standard. The mixture was vortexed for 5 s and then centrifuged at 12,000 rpm for 15 min at 4°C. The resulting supernatants were collected and analyzed using an Agilent LC system coupled with an AB SCIEX QTrap 6500+ mass spectrometer. TMAO and its related metabolites, including choline, betaine, and L-carnitine, were quantified.

### Statistical analysis

Statistical analyses were performed using GraphPad Prism (v9.0.0). Data are presented as mean ± standard error of mean (SEM). Data normality was evaluated using the Shapiro-Wilk test. All experiments were performed with at least three biological replicates (*n* ≥ 3). For comparisons between two groups, the Student’s two-tailed *t*-test was used for data following a normal distribution, while the Mann-Whitney U test was applied for non-normally distributed data. For comparisons involving three or more groups, one-way ANOVA followed by Tukey’s honestly significant difference (HSD) post hoc test was utilized for normally distributed data, and the Kruskal-Wallis test was employed for non-normally distributed data. Statistical significance was defined as *p < 0.05, **p < 0.01, ***p < 0.001, and ****p < 0.0001, with “ns” indicating no significant difference.

## Results

### Gut microbiota influences m^6^A RNA methylation in skeletal muscle

**Figure 1.**
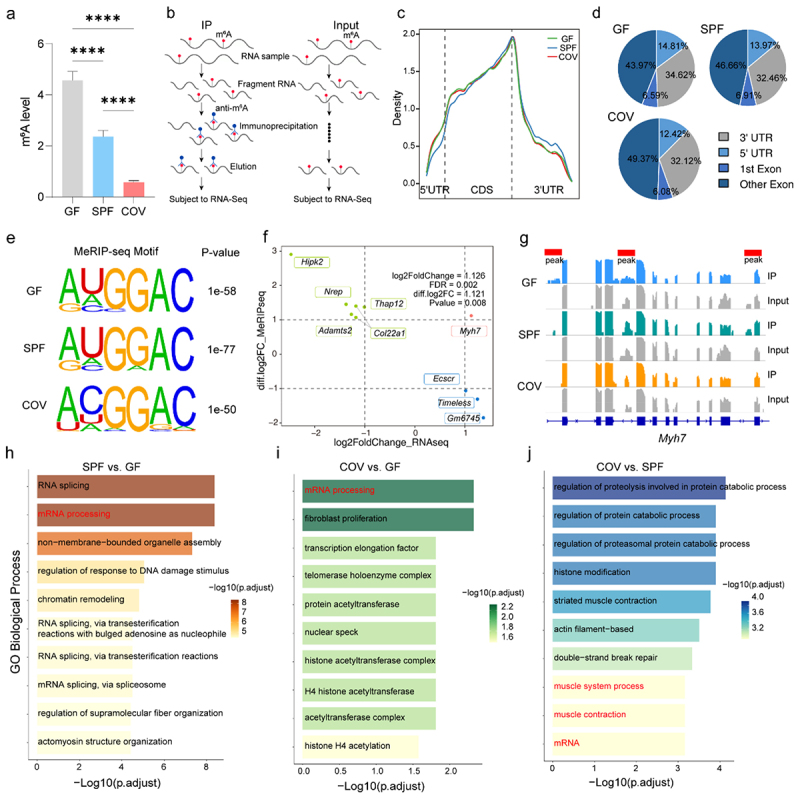
Gut microbiota regulates m^6^A RNA modification in skeletal muscle. (a) Quantification of the global m^6^A levels in GAS muscles from GF, SPF, and COV mice using ELISA (*n* = 3/group). (b) Schematic diagram of the MeRIP-seq workflow. (c) Density distribution of m^6^A peaks across transcript regions (including 5′-untranslated region (UTR), start codon, coding sequence (CDS), stop codon, and 3′-UTR) in GF, SPF, and COV mice (*n* = 3/group). (d) Genomic annotation of m^6^A peaks by transcript region (5‘-UTR, 1st exon, other exon, and 3‘-UTR) using ChIPseeker. (e) *De novo* motif analysis of m^6^A-enriched regions identified by HOMER. (f) Four-quadrant plot shows the distribution of genes with significant changes in both the RNA expression (|log_2_ FC| > 1 and FDR < 0.05) and m^6^A methylation (|log_2_ FC| > 1 and *p*-value < 0.05) levels, respectively. (g) IGV browser tracks views of the *Myh7* m^6^A peak (IP vs. Input) in GAS muscles from GF, SPF, and COV mice. (h-j) GO analysis of transcripts with differential m^6^A peaks. “mRNA processing” enriched in the SPF (h) and COV (i) compared to the GF groups, “muscle system process” and “muscle contraction” enriched in the COV compared to the SPF groups (j). Data are presented as the mean ± SEM. ****p < 0.0001, was calculated by two-sided Student’s *t*-test for two groups comparisons and by one-way ANOVA for three groups comparisons.

Given the reported interplay between gut microbiota and m^6^A RNA methylation,^23,24^ we hypothesized that gut microbiota regulates skeletal muscle phenotype via m^6^A RNA methylation. ELISA showed significantly elevated global m^6^A levels in the GAS muscles of GF mice versus the SPF and COV mice (reflecting distinct gut microbiota profiles; Supplementary Figure S2a-c), with SPF mice displaying higher m^6^A levels than COV mice (Figure 1a). Similarly, ABX-treated mice exhibited increased m^6^A levels in GAS muscles relative to controls (Supplementary Figure S2d). These results suggest that the gut microbiota affects m^6^A RNA methylation in skeletal muscle.

To identify microbiota-associated m^6^A modification involved in muscle fiber regulation, we performed MeRIP-seq on GAS muscles from GF, SPF, and COV mice (Figure 1b). Consistent with the canonical m^6^A distribution patterns,^25^ m^6^A peaks were enriched at the junction of the coding region (CDS) and 3’-untranslated region (3’-UTR, Figure 1c). The distribution of m^6^A peaks across four regions of transcripts (5’-UTR, 1^st^ exon, other exon, and 3’−UTR) was annotated in all three groups. The proportion of m^6^A peaks located in 3’-UTR and 5’-UTR regions showed minor variation (< 2%), but GF mice displayed more m^6^A peaks in 5’-UTR than either SPF or COV mice (Figure 1d). *De novo* motif analysis identified the conserved RRACH motif (*R* = A/G, H = A/C/U) across all groups (Figure 1e). Hierarchical clustering showed tighter clustering in the immunoprecipitation (IP) group compared to the input control (Supplementary Figure S2e). PCA revealed distinct group separations (Supplementary Figure S2f), indicating that gut microbiota markedly shapes the transcriptome-wide m^6^A methylation landscape in skeletal muscle.

Comparative analysis revealed 21 significantly gained and 101 significantly lost m^6^A peaks in the SPF mice compared to the GF mice, corresponding to 21 and 96 mRNA transcripts, respectively (Supplementary Figure S2g, h; Supplementary Table 2–1). In the COV vs GF comparison, 70 significant m^6^A peaks on 67 mRNA transcripts were gained, while 18 peaks on 17 mRNA transcripts were lost, respectively (Supplementary Figure S2g, h; Supplementary Table 2–2). Compared to SPF mice, 130 m^6^A peaks on 119 mRNA transcripts were gained and 43 peaks on 44 mRNA transcripts were lost in the COV group (|log_2_ FC| > 1 and *p* < 0.05; Supplementary Figure S2g, h; Supplementary Table 2–3). Integrating analysis of MeRIP-seq and RNA-seq data identified a hypermethylated peak (Chr14: 54988390–54988415) in *Myh7* mRNA (Figure 1f). The region had higher m^6^A deposition in SPF mice than COV mice (log_2_ FC = 1.12, *p* = 0.008), and an even higher peak in GF mice compared to SPF mice (Figure 1g; Supplementary Table 2). Compared to the GF mice, differentially expressed, m^6^A-enriched transcripts in SPF and COV groups were significantly involved in the GO term related to “mRNA processing” (Figure 1h,i). Between the SPF and COV mice, differentially methylated transcripts were enriched in pathways related to “muscle system process”, “muscle contraction”, and “mRNA” (Figure 1j). Therefore, these results implicate that the gut microbiota modulates muscle m^6^A RNA methylation and may thereby influence muscle fiber plasticity.

### Gut microbiota regulates skeletal muscle type transition by affecting Myh7 expression

**Figure 2.**
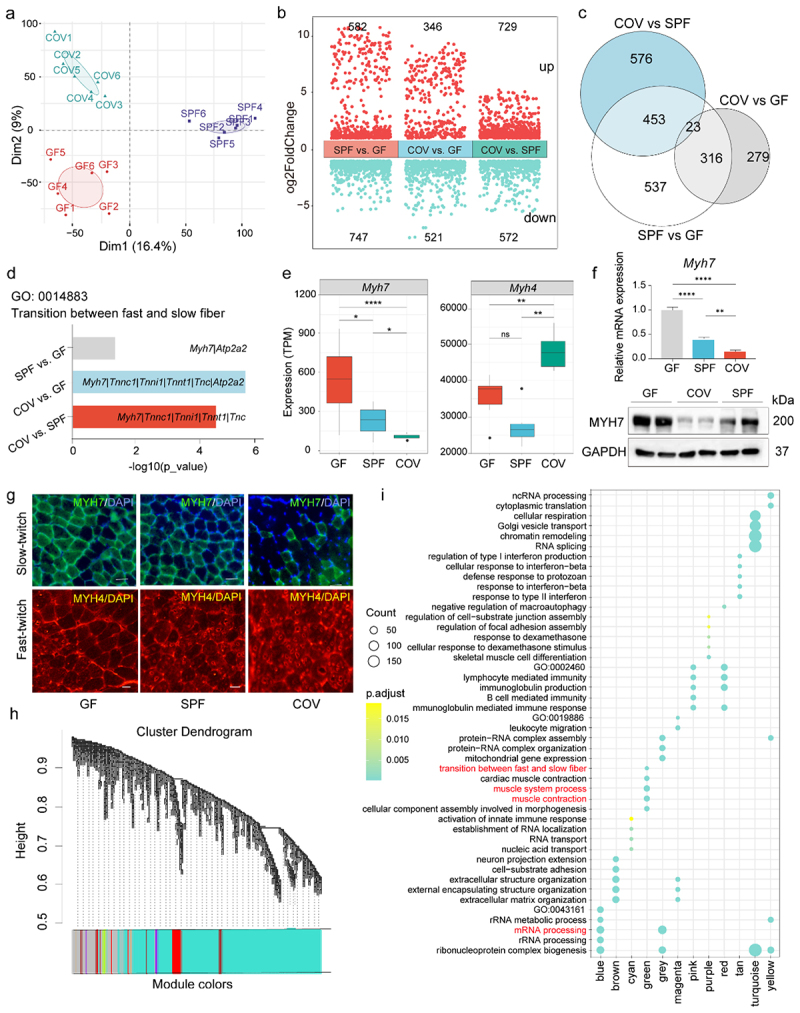
*Myh7* regulates skeletal muscle fiber transition in a gut microbiota manner. (a) PCA of transcriptomic profiles in GAS muscles among GF, SPF, and COV groups. (b) Volcano plots depicting DEGs in the GAS muscles among GF, SPF, and COV mice (|log_2_ FC| > 1, and FDR < 0.05). (c) Venn diagram showing the overlap of DEGs among the three pairwise comparisons. (d) GO enrichment analysis highlighting significant enrichment of DEGs in the biological process “transition between fast and slow fiber”. *Myh7* was the only gene consistently enriched across all comparisons. (e) Transcript quantification of per kilobase million (TPM) of *Myh7* and *Myh4* in GAS muscles. (f) RT-qPCR (top) and Western blot (bottom) analysis showing the *Myh7* mRNA and protein levels in GF, SPF, and COV mice. (g) Immunofluorescence staining of GAS muscle cross-sections for MYH7-positive (top) and MYH4-positive (bottom) fiber types. Scale bar: 20 μm. (h) WGCNA for gene clustering and module identification. (i) GO enrichment analysis of gene modules, with the green module enriched in the “transition between fast and slow fiber” pathway. Data are presented as the mean ± SEM. **p < 0.01, ***p < 0.001, ns indicates no significance, which was calculated by two-sided Student’s *t*-test for two groups comparisons and by one-way ANOVA for three groups comparisons.

To assess the effect of gut microbiota on gene expression in skeletal muscle, we performed RNA-seq analysis on the GAS muscles from GF, SPF, and COV mice. PCA revealed distinct clustering of transcriptomic profiles among the three groups (Figure 2a). Comparative analysis identified thousands of DEGs, with 1,329 DEGs between SPF and GF, 618 between COV and GF, and 1,052 between COV and SPF (Figure 2b,c). Among these DEGs, 23 genes, including *Myh7*, were commonly differentially expressed across all three pairwise group comparisons (Figure 2c; Supplementary Figure S3a). GO analysis revealed significant enrichment of DEGs in pathways associated with muscle fiber development, such as “regulation of supramolecular fiber organization” and “transition between fast and slow fiber” (Supplementary Figure S3b-d). Notably, the GO term “transition between fast and slow fiber” was significantly enriched in two of the three group comparisons (Figure 2d). In this term, *Myh7* was the only gene consistently enriched across all inter-group comparisons. Additionally, *Myh7* expression was highest in GF mice, followed by SPF and COV mice (Figure 2e,f). In contrast, *Myh4* displayed an opposing pattern, with the highest expression in COV mice and the lowest in SPF mice (Figure 2e).

Immunofluorescence assays revealed marked differences in muscle fiber-type composition among the three groups. Specifically, GF mice exhibited an increased proportion of MYH7-positive fibers and a reduction in MYH4-positive fibers compared to both SPF and COV mice, with SPF also showing a fiber-type shift relative to COV mice (Figure 2g; Supplementary Figure S3e). To further investigate the underlying molecular mechanisms involved in skeletal muscle fiber-type modulation, we performed WGCNA. A total of 12 gene modules were identified (Figure 2h). Notably, GO analysis revealed that the green module was significantly associated with the “transition between fast and slow fiber”, “muscle system process”, and “muscle contraction”, and the blue module was significantly enriched in genes related to “mRNA processing” (Figure 2i), adding further weight to our findings. Together, these results demonstrate that *Myh7* serves as a critical regulator of muscle fiber-type transition, modulated by the gut microbiota.

### Mettl3 promotes slow-type myofiber formation by regulating Myh7 in an m^6^A-Ythdf2-dependent manner

**Figure 3.**
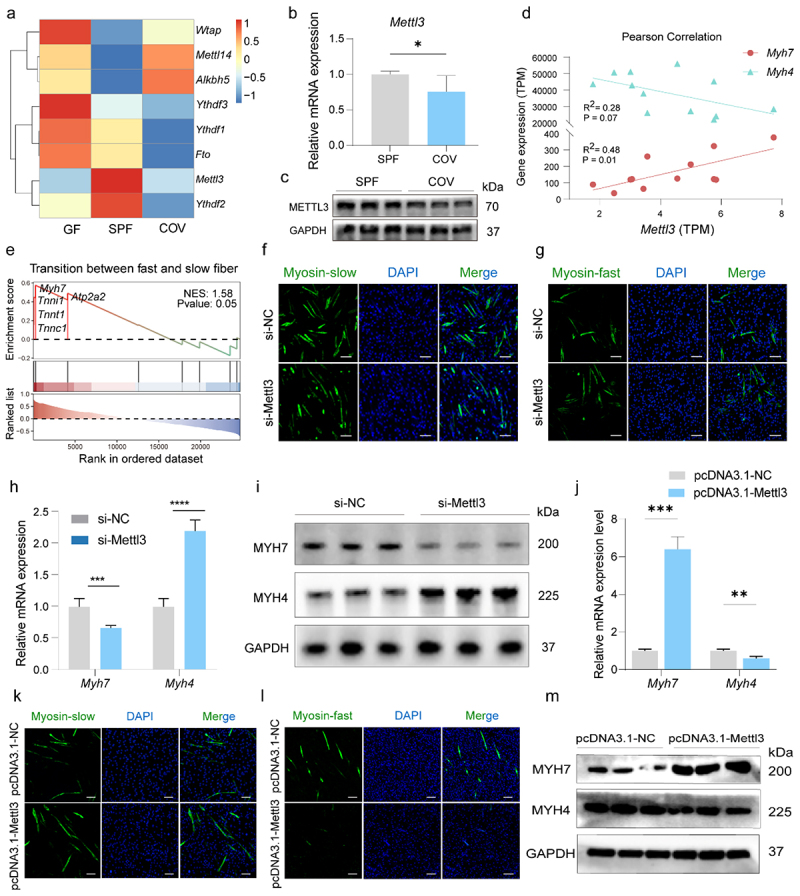
*Mettl3* mediates muscle slow fiber transition by regulating *Myh7* expression relying on gut microbiota. (a) Heatmap of RNA-seq profiles for core m^6^A methyltransferases in GAS muscles (*n* = 6/group). (b, c) RT-qPCR (b) and Western blot (c) analysis confirmed *Mettl3* mRNA and protein expression levels in SPF and COV groups (*n* = 3 per group). (d) Pearson correlation between *Mettl3* expression and the expression of *Myh7* and *Myh4*. (e) Single-gene GSEA of *Mettl3* in the RNA-seq of SPF and COV mice, showing positive enrichment of “transition between fast and slow fiber”. (f, g) Immunofluorescence assay of MYH7-positive (f) and MYH4-positive (g) fibers by *Mettl3* knockdown in the C2C12 myoblasts. Scale bar: 50 μm. (h, i) RT-qPCR (h) and Western blot (i) analysis of *Myh7* and *Myh4* mRNA and protein expression in the *Mettl3* knockdown and control groups. (j, k) Overexpression of *Mettl3* in the C2C12 myoblasts results in increased MYH7-positive fiber type (j) and decreased MYH4-positive fiber type (k), as assessed by immunofluorescence assay. Scale bar: 50 μm. (l, m) RT-qPCR (l) and Western blot (m) analysis of *Myh7* and *Myh4* mRNA and protein expressions in the pcDNA3.1-Mettl3 and control (pcDNA3.1-NC) groups. Data are presented as the mean ± SEM. *p < 0.05, **p < 0.01, ***p < 0.001, ****p < 0.0001, were calculated by two-sided Student’s *t*-test for two groups comparisons and by one-way ANOVA for three groups comparisons.

To delineate the role of m^6^A in muscle fiber switching, we quantified the expression of core m^6^A regulatory enzymes. Notably, the m^6^A “writer” *Mettl3* and the m^6^A “reader” *Ythdf2* exhibited higher expression in the SPF group, while the m^6^A demethylase *Alkbh5* was up-regulated in the COV group (Figure 3a). *Mettl3* expression was significantly higher at both mRNA and protein levels in the SPF than in COV mice (Figure 3b,c), and it showed a positive correlation with *Myh7* (*R*^*2*^ = 0.48, *p* = 0.01) and a negative correlation with *Myh4* (*R*^*2*^ = 0.28, *p* = 0.07) (Figure 3d). To explore potential pathways associated with *Mettl3*, we performed single-gene GSEA for *Mettl3* using RNA-seq data from SPF and COV mice. The analysis revealed significant enrichment in the GO term “transition between fast and slow fiber” (normalized enrichment score (NES) = 1.58, *p* = 0.05; Figure 3e). Functional validation in C2C12 myoblasts showed that *Mettl3* knockdown decreased the proportion of slow-twitch fibers while increasing fast-twitch fibers (Figure 3f,g), accompanied by reduced *Myh7* and increased *Myh4* expression at both mRNA and protein expression levels (Figure 3h,i). Conversely, *Mettl3* overexpression reversed this phenotype, enhancing the expression of slow-twitch marker *Myh7* and suppressing fast-twitch marker *Myh4* (Figure 3j-m). These results suggest *Mettl3* regulates muscle fiber composition through m^6^A modification of *Myh7*, in a gut microbiota-dependent manner.

**Figure 4.**
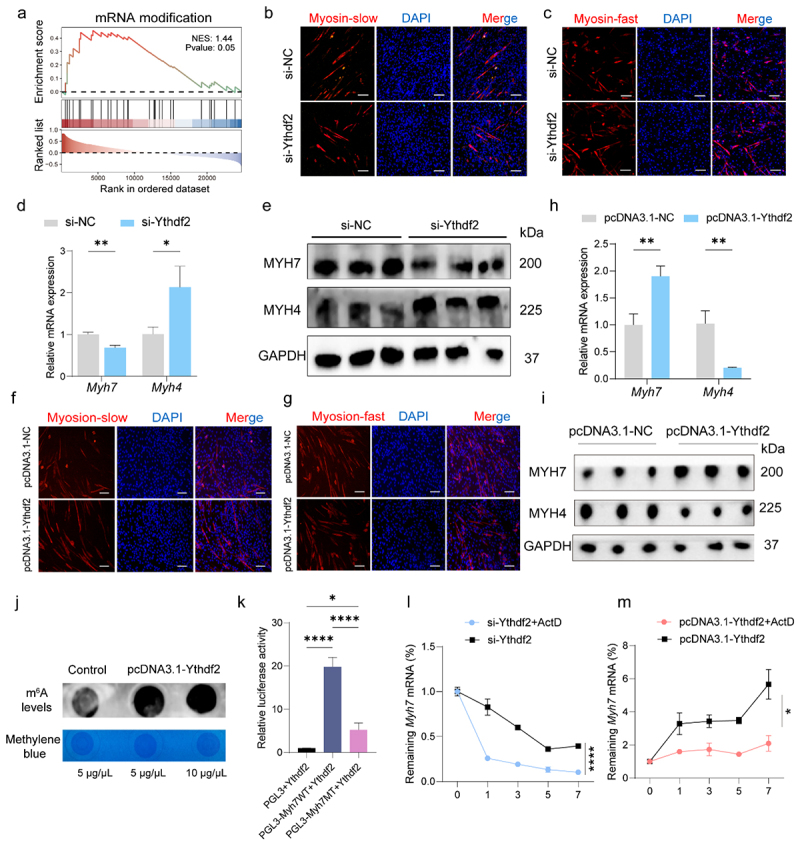
*Ythdf2* regulates *Myh7* expression during myofiber type transition in an m^6^A-dependent manner. (a) Single-gene GSEA of *Ythdf2* showing “mRNA modification” enrichment in GAS muscles from SPF and COV mice. (b, c) Immunofluorescence assay of MYH7-positive (b) and MYH4-positive (c) fiber types following *Ythdf2* knockdown in C2C12 differentiated myoblasts. Scale bar: 50 μm. (d, e) RT-qPCR (d) and Western blot (e) analysis of *Myh7* and *Myh4* mRNA and protein expressions in *Ythdf2* knockdown. (f, g) Immunofluorescence assay of MYH7-positive (f) and MYH4-positive (g) fiber types following *Ythdf2* overexpression in C2C12 myoblasts. Scale bar: 50 μm. (h-i) RT-qPCR (h) and Western blot (i) analysis of *Myh7* and *Myh4* mRNA and protein expression in *Ythdf2* overexpression and control groups. (j) Dot blot assay demonstrating that *Ythdf2* overexpression increased global m^6^A levels at two concentrations (5 and 10 µg/μL), with methylene blue staining as a loading control. (k) Dual-luciferase reporter assay confirming *Ythdf2* binding to *Myh7* mRNA. (l, m) RT-qPCR-based mRNA decay analysis of *Myh7* stability in the C2C12 cells transfected with si-Ythdf2 and si-NC (l) and pcDNA3.1-Ythdf2 and pcDNA3.1-NC (m), followed by ActD treatment (5 µg/mL) at indicated time points. Data are presented as the mean ± SEM. *p < 0.05, **p < 0.01, ***p < 0.001, ****p < 0.0001, which was calculated by two-sided Student’s *t*-test for two groups comparisons and by one-way ANOVA for three groups comparisons.

Given that METTL3 regulates myogenesis via the m^6^A-YTHDF2 signaling axis,^27^ and YTHDF2 governs skeletal muscle growth and postnatal muscle size,^38^ we further investigated the function of YTHDF2 in fiber-type switching. Single-gene GSEA highlighted a strong enrichment of GO terms associated with “mRNA modification” in relation to *Ythdf2* expression (NES = 1.44, *p* = 0.05, Figure 4a). Knockdown of *Ythdf2* phenocopied the effects of *Mettl3* knockdown, promoting a fast-twitch fiber phenotype (Figure 4b-e), while *Ythdf2* overexpression favored slow-twitch fiber formation, mimicking the effects of *Mettl3* overexpression (Figure 4f-i). Moreover, m^6^A dot blot analysis showed that *Ythdf2* overexpression increased total m^6^A methylation levels (Figure 4j). To validate the specificity of *Ythdf2*-mediated recognition of *Myh7* mRNA, a dual luciferase assay confirmed that mutating the classic m^6^A motif (RRACH) reduced *Ythdf2* binding to the *Myh7* transcript (Figure 4k). RNA stability assays further revealed that *Ythdf2* knockdown accelerated the degradation of *Myh7* mRNA, while its overexpression enhanced *Myh7* transcript stability (Figure 4l,m). Additionally, in the ABX-treated mice, both *Mettl3* and *Ythdf2* expression were increased (Supplementary Figure S4a,b), further supporting a link between gut microbiota and the m^6^A regulatory axis. Altogether, these results uncover an intricate regulatory network involving *Mettl3*, *Ythdf2*, and *Myh7*, whereby m^6^A-dependent mechanisms govern skeletal muscle fiber-type remodeling in response to gut microbiota.

### Microbiota-derived betaine is a candidate metabolite regulating skeletal muscle fiber remodeling

**Figure 5.**
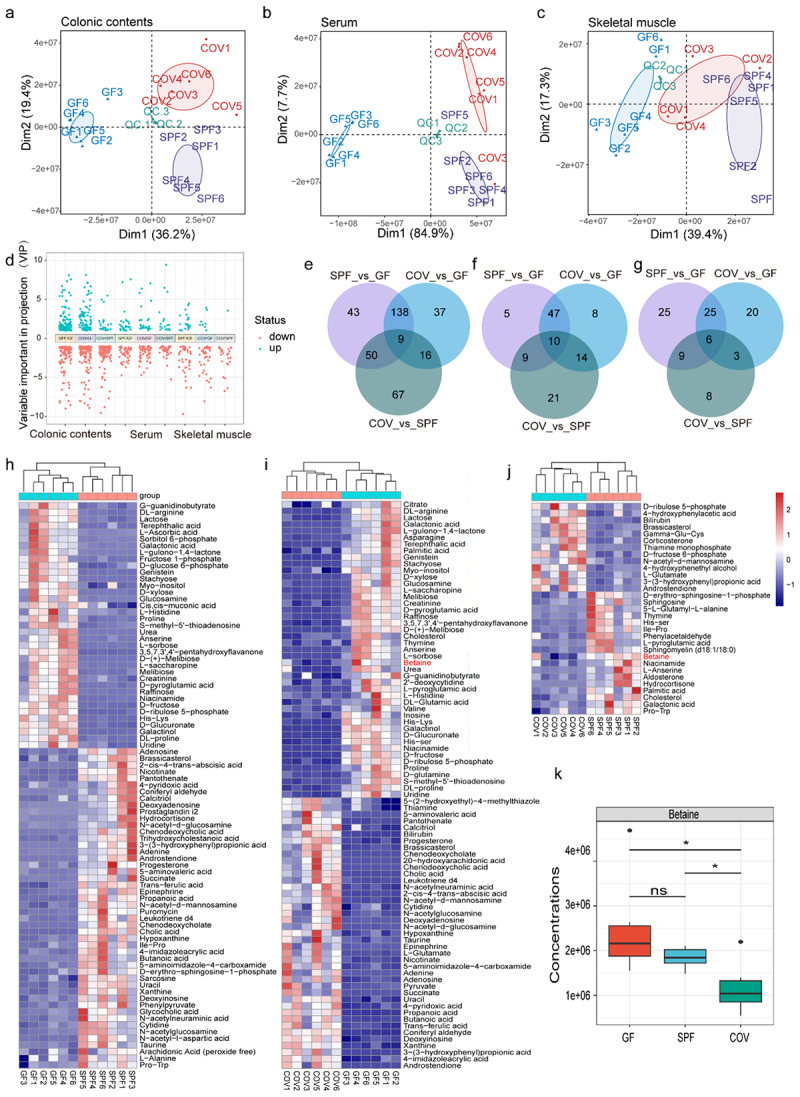
Microbiota-derived betaine is a promising metabolite modulating skeletal muscle fiber remodeling. (a-c) PCA of metabolite contents by LC-MS/MS in colonic contents (a), serum (b), and GAS muscles (c) from GF, SPF, COV mice, and quality control (QC) samples (*n* = 6/group for the colon and serum, *n* = 6, 6, 4 for the GAS muscles). (d) Volcano plots displaying DEMs in colonic contents (left), serum (medium), and GAS muscles (right) among GF, SPF, and COV mice (VIP scores > 1 and *p* < 0.05). (e-g) Venn plots illustrating overlapped DEMs among the GF, SPF, and COV mice in the colonic contents (e), serum (f), and GAS muscles (g). (h-j) Heatmaps display the DEMs enriched in KEGG “metabolic pathway” of colonic contents in comparisons between SPF and GF mice (h), COV and GF mice (i), and COV and SPF mice (j). (k) Box plot showing the levels of betaine were significantly different in the colonic contents among the GF, SPF, and COV mice, in which displays median (center line), 75th (upper limit of box) and 25th percentiles (lower limit of box) and outliers (whiskers) if values do not exceed 1.5 × interquartile range. *p < 0.05, ns indicates no significance, by two-sided Student’s *t*-test for two groups comparisons and by one-way ANOVA for three groups comparisons.

Untargeted metabolomic profiling of colonic contents, serum, and skeletal muscle from GF, SPF, and COV mice revealed distinct tissue-specific metabolic signatures, as illustrated by PCA (Figure 5a-c). Pairwise comparisons identified hundreds of differentially expressed metabolites (DEMs, VIP scores > 1 and *p* < 0.05, Figure 5d). In the colonic contents, 137 metabolites were upregulated and 103 downregulated in SPF versus GF mice, while COV versus GF yielded 90 upregulated and 110 downregulated DEMs. The COV versus SPF comparison demonstrated 26 upregulated and 116 downregulated DEMs. In serum, SPF versus GF mice exhibited 27 upregulated and 44 downregulated DEMs, COV versus GF had 26 upregulated and 53 downregulated, and COV versus SPF showed 18 upregulated and 36 downregulated metabolites. Skeletal muscle analysis revealed 24 upregulated and 41 downregulated DEMs in SPF versus GF, 9 upregulated and 45 downregulated DEMs in COV versus GF, and 1 upregulated and 25 downregulated DEMs in COV versus SPF comparisons. Overlapping DEMs across group comparisons revealed 9, 10, and 6 shared metabolites in the colonic contents, serum, and skeletal muscle, respectively (Figure 5e-g, Supplementary Table 3–1 ~ 9). KEGG pathway enrichment showed that these DEMs were significantly mapped to “metabolic pathways” (mmu01100) in colonic content across GF/SPF (Figure 5h), GF/COV (Figure 5i), and SPF/COV groups (Figure 5j). These metabolic signatures reflect an interconnected biochemical network, underscoring active gut-muscle axis communication.

Interestingly, betaine, a regulator of skeletal muscle slow-twitch fiber formation^17^ and a key methyl donor, was notably more abundant in the colonic contents of the SPF mice compared to the COV mice (Figure 5k). As a methyl donor,^15,16^ we proposed that gut microbiota may regulate betaine to influence muscle fiber conversion through m^6^A RNA methylation. Besides, DEMs enriched in “metabolic pathways” were detected in the serum among GF/SPF (Supplementary Figure S5a), GF/COV (Supplementary Figure S5b), and SPF/COV groups (Supplementary Figure S5c), as well as skeletal muscle among GF/SPF (Supplementary Figure S5d), GF/COV (Supplementary Figure S5e), and SPF/COV groups (Supplementary Figure S5f). Betaine levels were highest in the skeletal muscle of GF mice (Supplementary Figure S5g) and a significant positive correlation was observed between betaine levels in colonic contents and skeletal muscles (*R*^*2*^ = 0.40, *p* = 0.0091; Supplementary Figure S5h). These findings indicate that betaine may originate from gut microbial metabolism and enter circulation to reach peripheral tissues such as skeletal muscle. Furthermore, carnitine, being metabolized to betaine by gut microbiota, was more abundant in GF mice compared to SPF and COV mice (Supplementary Table 3). Choline, irreversibly converted to betaine,^39^ was higher in COV, displaying an inverse trend relative to betaine levels (Supplementary Figure S5i). The levels of creatine and betaine in colonic contents, along with betaine and choline in skeletal muscle, may reflect a dynamic metabolic balance among these interrelated compounds.^40^ Overall, these findings implicate that microbiota-derived betaine is a critical regulator of muscle fiber transition, potentially through methylation-dependent mechanisms such as m^6^A RNA modification.

### Betaine contributes to skeletal muscle fiber transition via m^6^A RNA methylation

**Figure 6.**
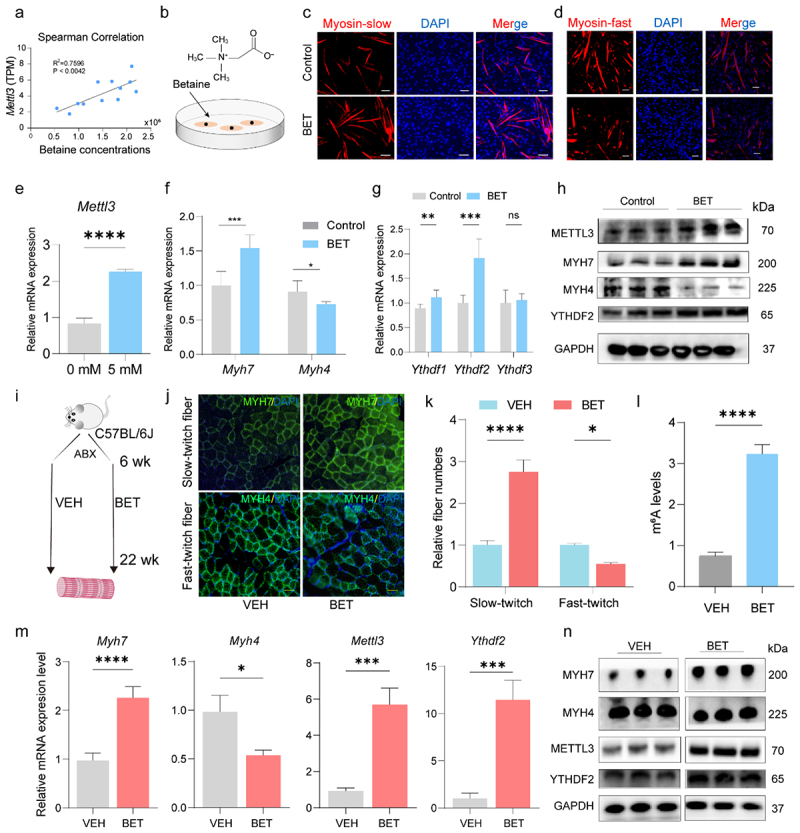
Betaine supplementation contributes to skeletal muscle fiber conversion via m^6^A RNA methylation. (a) Spearman correlation between betaine concentration and *Mettl3* expression. (b) Schematic diagram of betaine treatment in differentiated C2C12 myoblasts. (c,d) Immunofluorescence assay of MYH7-positive (c) and MYH4-positive fiber types (d) following betaine treatment. Scale bar: 50 μm. (e-g) RT-qPCR analysis of *Mettl3* (e), *Myh7*, and *Myh4* (f), *Ythdf1*, *Ythdf2*, *Ythdf3* (g) mRNA expression after betaine stimulation. (h) Western blot of METTL3, MYH7, MYH4, and YTHDF2 protein levels with GAPDH as the loading control in betaine-treated cells. (i) Scheme of betaine treatment *in vivo* (*n* = 10/group). (j) Immunofluorescence assay of GAS muscle cross-sections for MYH7-positive (top) and MYH4-positive (bottom) fiber types in vehicle (VEH), and betaine (BET) mice. Scale bar: 20 μm. (k) Quantification of the number of muscle fiber types in the VEH and BET groups. (l) Global m^6^A levels in GAS muscles from the VEH, and BET mice. (m) RT-qPCR analysis of *Myh7*, *Myh4*, *Mettl3*, and *Ythdf2* mRNA expression in GAS muscles. (n) Western blot of MYH7, MYH4, METTL3, and YTHDF2 protein expression in GAS muscles upon *in vivo* betaine treatment; GAPDH used as internal control. Data are presented as the mean ± SEM. *p < 0.05, **p < 0.01, ***p < 0.001, ****p < 0.0001, ns indicates no significance, which was calculated by two-sided Student’s *t*-test for two groups comparisons and by one-way ANOVA for three groups comparisons.

Betaine levels showed a strong positive correlation with *Mettl3* expression in GAS muscles (*R*^*2*^ = 0.80, *p* = 0.0042, Figure 6a), suggesting that betaine may regulate muscle fiber-type switching via *Mettl3*. In C2C12 myoblasts, treatment with 5 mM betaine during differentiation (Figure 6b) significantly increased the proportion of MYH7-positive cells (Figure 6c), and decreased the proportion of MYH4-positive cells (Figure 6d). Betaine stimulation also elevated the expression of *Myh7*, *Mettl3*, and *Ythdf2*, while suppressing *Myh4* expression at both mRNA (Figure 6e-g) and protein levels (Figure 6h).

For *in vivo* validation, 10 mM betaine was administered in drinking water to the C57BL/6J mice for 16 weeks (Figure 6i; Supplementary Figure S6a). Immunofluorescence assay of the GAS muscles showed that the betaine-treated (BET) group exhibited a significantly greater number of slow-twitch fibers and fewer fast-twitch fibers compared to the vehicle (VEH) group (Figure 6j,k). No significant differences in body weight or skeletal muscle mass were observed between the two groups, except for a decrease in the tibialis anterior muscle mass in the BET group (Supplementary Figure S6b). Global m^6^A RNA methylation levels were significantly higher in the BET group than in the VEH group (Figure 6l). *Myh7* expression was increased, while *Myh4* expression was decreased in the BET group compared to the VEH group, at both mRNA (Figure 6m) and protein levels (Figure 6n). Taken together, these findings demonstrate that betaine contributes to skeletal muscle fiber transition via the betaine-*Mettl3*-m^6^A-*Myh7* axis, highlighting a microbiota-derived metabolite–epigenetic pathway underlying muscle fiber plasticity.

### Gut microbiota orchestrating betaine modulates muscle fiber conversion via m^6^A RNA methylation

**Figure 7.**
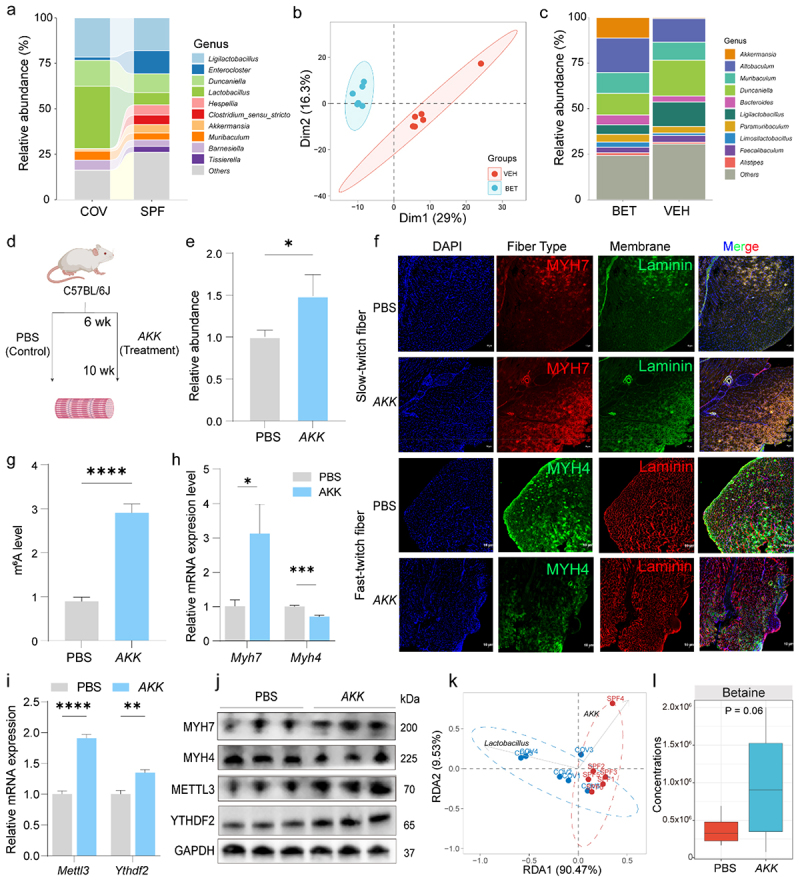
*AKK* orchestrating betaine modulates skeletal muscle fiber characteristics via m^6^A RNA methylation. (a) Relative abundance of the top 10 bacterial genera in colonic contents between the SPF and COV groups. (b) PCA of gut microbiota profiles based on 16S rRNA gene sequencing in VEH and BET mice (*n* = 6/group). (c) Genus-level comparison of the top 10 microbial taxa between VEH and BET groups (*n* = 6/group). (d) Schematic of *AKK* transplantation in mice compared to the control (PBS) group *in vivo*. (e) Relative abundance of *AKK* in the *AKK*- and PBS-treated mice as assessed by RT-qPCR. (f) Immunofluorescence images showing increased MYH7-positive fiber-type and decreased MYH4-positive fiber-type in *AKK*-treated mice. Scale bar: 10 μm. (g) Global m^6^A levels in GAS muscles of the *AKK*-treated versus PBS-treated group. (h-j) RT-qPCR (h, i) and (j) Western blot analyses demonstrating increased expression of *Myh7*, *Mettl3*, and *Ythdf2* and decreased *Myh4* expression in the *AKK* group. (k) RDA revealed that *AKK* and *lactobacillus* were positively and negatively associated, respectively, with DEMs in “metabolic pathways” between COV and SPF mice. (l) Box plots showing increased betaine levels in the colonic contents between *AKK*-treated mice compared to PBS-treated controls, in which displays median (center line), 75th (upper limit of box) and 25th percentiles (lower limit of box) and outliers (whiskers) if values do not exceed 1.5 × interquartile range. Data are presented as the mean ± SEM. *p < 0.05, **p < 0.01, ***p < 0.001, ****p < 0.0001, ns indicates no significance, was calculated by two-sided Student’s *t*-test for two groups comparisons and by one-way ANOVA for three groups comparisons.

To investigate the relationship between gut microbiota and muscle fiber composition, we compared the microbial profiles of SPF and COV mice. In the comparison of the different mouse groups, we performed an alpha-diversity analysis to assess the microbial diversity within each group. Our results showed higher alpha-diversity in SPF than COV mice (Supplementary Table 4–1). At the phylum level, *Bacteroidetes* and *Firmicutes* were the dominant taxa in both groups (Supplementary Figure S7a; Supplementary Table 5–1). Genus-level analysis revealed that beneficial microbes, such as *AKK* and *Bifidobacterium*, were significantly more abundant in SPF mice (4.67%, and 2.21%) compared to COV mice (1.07%, and 0.02%). In contrast, *Lactobacillus* was higher in COV mice (34.36%) than SPF (6.74%) mice (Figure 7a; Supplementary Table 5–2). After taxonomic assignment, the OTU table was input into PICRUSt2 to predict the functional gene profiles using KEGG pathways. The results displayed that the pathways related to “G protein-coupled receptors”, “cell growth”, and “Neuroactive ligand-receptor interaction” were significantly enriched in SPF mice (Supplementary Figure S7b). In betaine-treated mice, PCA (Figure 7b) and ANOSIM (*R* = 0.98, *p* = 0.005; Supplementary Figure S7c) showed distinct microbial community structures between the BET and VEH groups, while alpha-diversity showed no significant difference (Supplementary Table 4–2). At the phylum level, *Bacteroidetes* and *Firmicutes* remained dominant across both groups (Supplementary Figure S7d; Supplementary Table 5–3). Notably, *AKK* was significantly increased in the BET group (10.03%) compared to the VEH group (0.58%) (Figure 7c; Supplementary Table 5–4). In addition, several KEGG pathways, including “lipid metabolism” and “steroid biosynthesis”, were significantly enriched in the BET group (Supplementary Figure S7e).

Given the established role of *AKK* in skeletal muscle function, we assessed its direct effect on muscle fiber-type transition. Following ABX treatment, mice were subjected to daily oral gavage with *AKK* or PBS for 30 consecutive days (Figure 7d). Oral gavage with *AKK* markedly increased its colonization in feces (Figure 7e), accompanied by an increased proportion of slow-twitch fibers and a decreased proportion of fast-twitch fibers (Figure 7f). *AKK*-treated mice significantly increased the global m^6^A RNA methylation levels (Figure 7g), along with upregulation of *Myh7*, *Mettl3*, and *Ythdf2* expression, and downregulation of *Myh4* at both mRNA (Figure 7h-i) and protein levels (Figure 7j). RDA indicated that a positive correlation between *AKK* and DEMs-enriched in “metabolic pathways”, including betaine, while *Lactobacillus* exhibited a negative association (Figure 7k). TMAO targeted metabolomics further revealed higher betaine levels in the colonic contents of the *AKK*-administered mice than in the PBS group (Figure 7l). Together, these findings echo the effects of *AKK* colonization coordinating betaine in regulating skeletal muscle fiber transition via the gut microbiota–m^6^A–*Myh7* axis.

## Discussion

Skeletal muscle fiber composition is a critical determinant of muscle quality and metabolic function.^41^ Accumulating evidence indicates that gut microbiota modulates various skeletal muscle traits, including muscle mass, sarcopenia,^42–44^ and muscle fatty acid deposition and meat quality in livestock.^45,46^ In this study, we demonstrate that gut microbes influence skeletal muscle fiber transition by elevating the methyl donor metabolite betaine, enhancing m^6^A RNA methylation, and specifically upregulating *Myh7* expression in a murine model. This discovery uncovers a novel signaling axis linking gut microbes to muscle fiber plasticity.^39,47–49^ Consistently, pigs and mice receiving FMT exhibit increased *Myh7* expression and a higher proportion of slow-twitch fibers,^11^ validating our mechanistic findings.

In m^6^A methylation, *Mettl3* regulates muscle fiber transition, consistent with its role in promoting muscle growth,^25,26^ regulating muscle stem cell and myoblast state transitions,^50^ and maintaining muscle homeostasis.^51^ Importantly, *Mettl3* expression was altered by gut microbiota, influencing *Myh7* expression and remodeling muscle fiber types, thereby demonstrating a microbiota-dependent mechanism of m^6^A modification. In parallel, the m^6^A reader *Ythdf2* functions as a mediator of muscle fiber transition by modulating *Myh7* expression. Specifically, *Ythdf2* directly recognizes and binds to the m^6^A site on *Myh7*, enhancing its stability and transcription. Similarly, in cardiomyocytes, *Ythdf2* alleviates cardiac hypertrophy by modulating *Myh7* mRNA stability, rather than mRNA transition,^35^ highlighting a context-specific role of *Ythdf2*. Therefore, the coordinated action of *Mettl3* and *Ythdf2* with *Myh7* is pivotal for directing skeletal muscle fiber switching. The deposition of m^6^A RNA modification, recognized by m^6^A-binding proteins, is essential for muscle myogenesis.^27,52^ Moreover, gut microbiota regulates the landscape of host transcriptome and m^6^A epitranscriptome via the bile acid metabolism pathway,^53^ which aligns with our current results. Here, our evidence highlighted m^6^A RNA methylation modification as a novel epigenetic mechanism mediating the crosstalk between gut microbiota and skeletal muscle.

Furthermore, betaine, a methyl-donor metabolite involved in one-carbon metabolism, modulates muscle fiber conversion, consistent with findings in both humans^54^ and mice.^17,18^ Betaine correlates with increased type I (slow-twitch) fibers, and regulates glucose and lipid metabolism in mice.^18^ Clinically, lower plasma betaine levels in humans have been linked to elevated risks of metabolic disorders, including insulin resistance, coronary artery disease, and obesity.^55^ Functionally, betaine serves as a substrate in methionine synthesis, generating S-adenosylmethionine (SAM).^56,57^ SAM donates methyl groups required for both DNA methylation^58,59^ and m^6^A RNA methylation.^60,61^ Similar to previous findings, betaine has hepatoprotective effects referring to *FTO*-independent function,^20^ and exerts beneficial effects on impaired adipose tissue function through m^6^A-dependent pathways.^61^ While dietary sources and supplements contribute to betaine levels, gut microbiota metabolizes dietary choline and carnitine into betaine via the complex metabolic network.^14^

Our results indicate that microbiota-derived betaine promotes fast-to-slow fibers in skeletal muscle. Previous studies reported that levels of alanine betaine and 5-aminovaleric acid betaine are significantly lower in the intestines of the GF compared to COV mice.^14^ In line with this, we observed that altered levels of betaine in GF versus non-GF mice, along with reciprocal changes in choline, the precursor of acetylcholine essential for neuromuscular junction function, further support the role of gut microbiota in betaine biosynthesis.^14,15^ These findings align with evidence that carnitine from *Lactobacillus reuteri* improves meat quality by affecting slow- and fast-fiber composition in pigs,^45^ and that microbiota-associated nicotinamide attenuated amyotrophic lateral sclerosis in mice,^62^ both of which indirectly support a role for microbiota-derived betaine in muscle fiber-type transition. In terms of microbiota functional predictions, “lipid metabolism” pathway was significantly enriched in the BET group, which may align with the known relationship between betaine supplementation and anti-obesity effects.^18^ Here, our results identify betaine as a novel microbiota-derived key metabolite that orchestrates fiber-type remodeling via the betaine-*Mettl3*-m^6^A-*Myh7* axis.

Regarding *AKK* functions, it is established as a beneficial gut microbe enriched in lean phenotypes,^63^ and negatively correlated with obesity.^64,65^ It has been shown to reshape the gut microbiota,^66^ modulate metabolic signaling,^67^ and regulate the immune system.^68^ In our study, betaine supplementation increased *AKK* abundance, consistent with previous reports.^18^ Our results reveal a novel role for *AKK* in regulating skeletal muscle fiber transition, similar to its ability to restore the fiber size,^69^ and attenuate amyotrophic lateral sclerosis by increasing nicotinamide levels.^62^ Also, *AKK* levels decline in aging mice,^69^ and its supplementation remodels muscle fiber composition by activating the m^6^A-*Myh7* axis. SCFAs are known mediators of the gut-muscle axis,^10,13,47^ and in our functional analysis, pathways related to “G protein-coupled receptors”, “cell growth”, and “neuroactive ligand-receptor interaction” were enriched in SPF mice. These pathways may coordinate with “metabolic pathways” to influence skeletal muscle remodeling. Thus, these findings highlight *AKK* as a promising microbial modulator of skeletal muscle fiber plasticity and muscle homeostasis.^70^

Given the importance of microbiota-derived metabolites, our findings further show that *AKK* enhances betaine metabolic flux in murine models, aligning with prior reports on betaine-*AKK* symbiosis.^14^ Notably, although *AKK* does not produce betaine *de novo*, it modulates its bioavailability through two key mechanisms: (1) by competing with other microbes for choline consumption; and (2) by degrading mucin to stimulate betaine-producing commensals.^71^ Through these mechanisms, *AKK* contributes to the regulation of intestinal betaine levels,^14^ promotes the production of bioactive metabolites, modulates the serum metabolome,^72^ and stimulates SCFA-producing microbes (e.g., acetate and butyrate). A few microorganisms (including bacteria, archaea, and fungi) can synthesize betaine through a two-step oxidation of choline via the intermediate betaine aldehyde using two enzymes (choline dehydrogenase and betaine aldehyde dehydrogenase).^15^ As reviewed, heterotrophic bacteria, including *Escherichia coli*, *Bacillus subtilis*, and *Arthrobacter globiformis*, are capable of *de novo* betaine synthesis.^15^ In turn, betaine treatment improves gut microbial ecology, enhancing SCFAs fermentation, and exerting anti-obesity and metabolic syndrome-alleviating effects in mice.^15,18,73^ These beneficial bacteria contain *AKK*, *Prevotella*, *Ruminococcus*, *Oscillospira*, *Bifidobacterium*, *Lactobacillus*, and *Dorea*.^18^ Despite strong associations between *AKK* and betaine metabolism, causality remains unresolved, largely due to: (1) microbial functional redundancy (e.g., *Prevotella* compensates for betaine regulation),^71^ and (2) context-dependent responses (e.g., dietary ratios of choline to betaine alter microbial consortium system).^73^ To dissect causal mechanisms, future studies should employ *AKK*-specific knockout microbiota models combined with isotopic tracing (e.g., ^13^C-betaine). Collectively, these findings support the concept that *AKK* and betaine engage in cross-kingdom metabolic crosstalk, constituting a microbiome-metabolome axis essential for host metabolic health.

**Figure 8.**
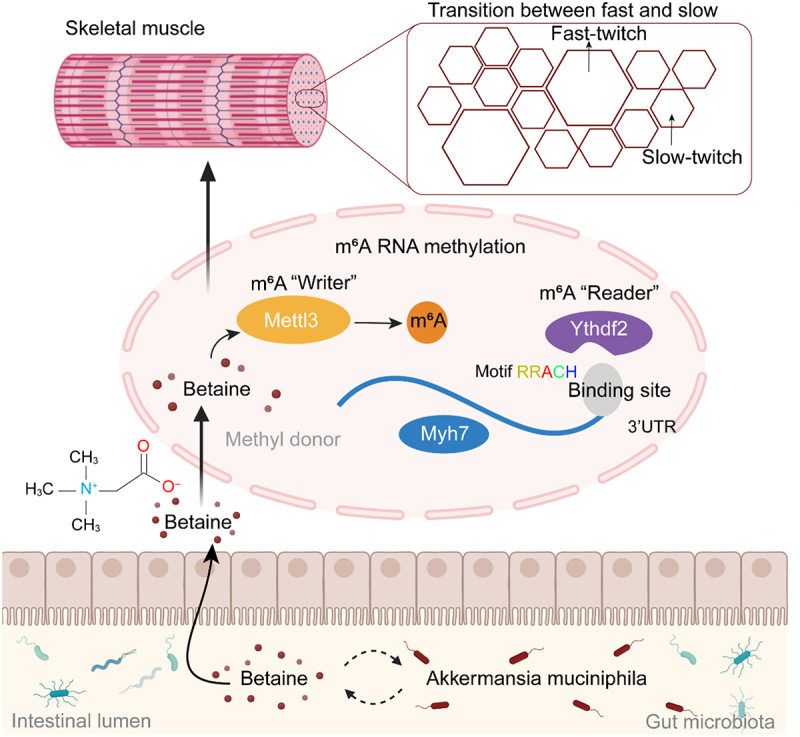
The mechanism of gut microbiota orchestrating betaine regulates skeletal muscle fiber via m^6^A RNA methylation and *Myh7* expression in the gut-muscle axis (created with BioRender.com).

In conclusion, our study revealed that muscle fiber type composition is intricately regulated by the gut-muscle axis. Epigenetically, *AKK* and betaine enhance m^6^A methylation levels by modulating the “writer” *Mettl3* and “reader” *Ythdf2*, affecting *Myh7* expression and stability, thereby facilitating the skeletal muscle fiber conversion (Figure 8). These findings establish a functional gut microbiota–metabolite–epigenetic–muscle axis that orchestrates muscle plasticity. Subsequent researches are warranted to explore additional gut microbes and metabolites that influence skeletal muscle fiber reprogramming. A limitation of the current study is a bias toward female mice, which presents an opportunity for future studies to induce male mice, ensuring broader applicability and investigating potential sex-based differences in gut-muscle interactions. Overall, these findings offer novel insights into the underlying mechanisms of the gut-muscle axis, lay the groundwork for microbiota- and metabolite-targeted interventions aimed at improving muscle health, and highlight their significance in preventing muscle degeneration and dysfunction.

## Supplementary Material

Supplementary Tables.zip

Supplementary_Figures_R2 clean.docx

## Data Availability

The data that support the findings of this study have been deposited into the CNGB Sequence Archive (CNSA) of China National GeneBank Database (CNGBdb) with accession number: CNP0005639 and CNP0005541. The remaining data are available within the article, supplementary information, or upon reasonable request from the correspondence.
