# Supplementary material for: Gut microbiota-mediated betaine regulates skeletal muscle fiber type transition by affecting m6A RNA methylation and Myh7 expression
Source: Gut Microbes. 2025 Aug 18;17(1):2545434. doi: 10.1080/19490976.2025.2545434 (PMC12363516; doi:10.1080/19490976.2025.2545434)
Supplement: Supplementary_Figures_R2 clean.docx [file KGMI_A_2545434_SM9026.docx]

**
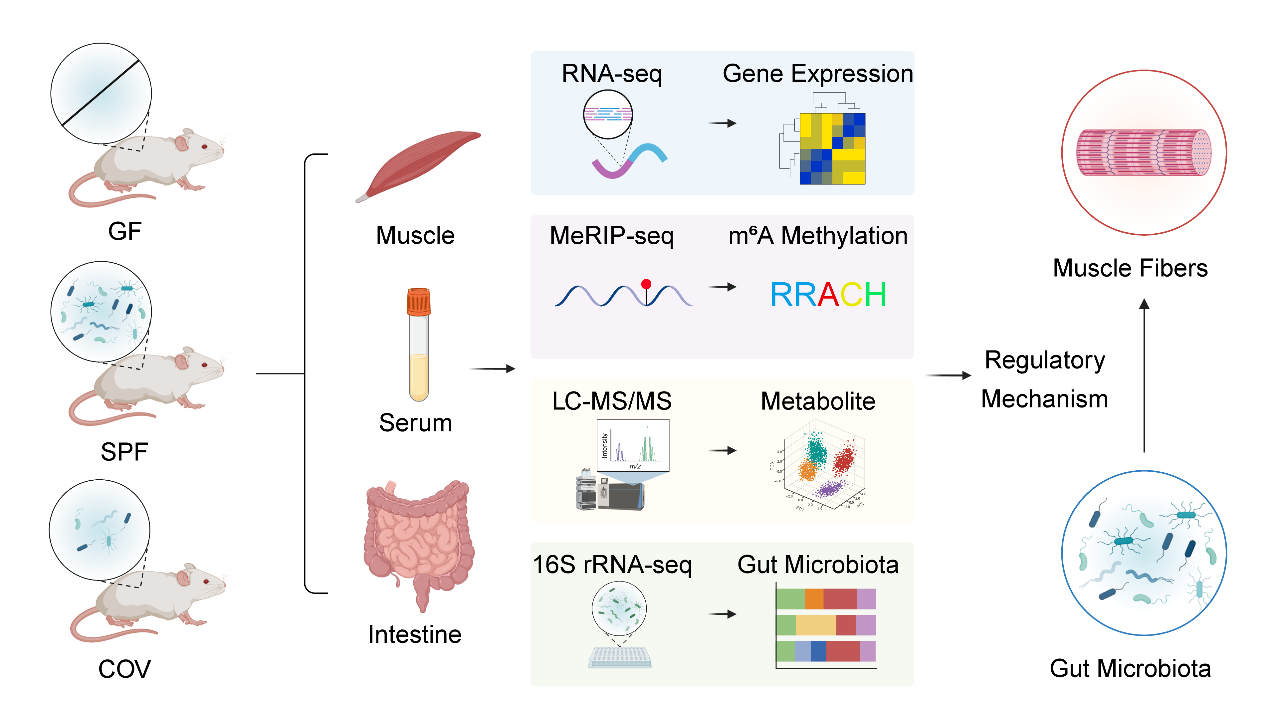
Supplementary Figures**

**Supplementary Figure 1.** The experimental design of the gut microbiota influences m^6^A modification in the skeletal muscle (Created with BioRender.com.).

**
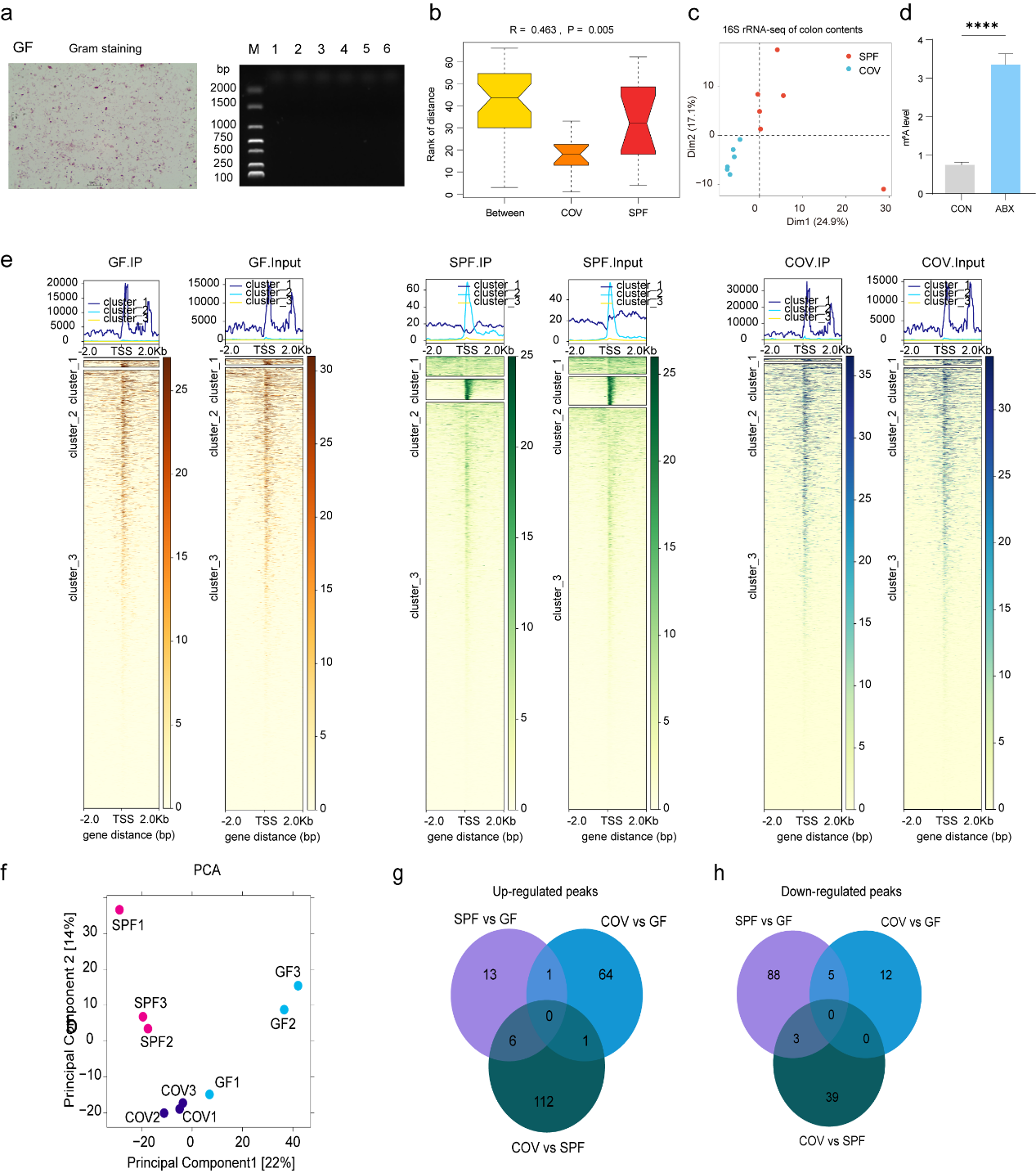
Supplementary Figure 2.** Gut microbiota regulates m^6^A modification in skeletal muscle. (a) Representative images of gram-stained slides and PCR examination of fecal samples from GF mice, showing the absence of bacteria, with most debris not retaining the Gram stain. (b) ANOISM and (c) PCA of bacterial 16S rRNA gene sequencing data reveal distinct microbial compositions in the colonic contents between SPF and COV groups. (d) Global levels of m^6^A of the GAS tissues from ABX and CON mice by ELISA (n = 3/group). (e) Profile and heatmap showing the cluster in the IP were higher than the input group. (f) PCA of MeRIP-seq shows clear separation among GF, SPF, and COV mice, analyzed using MACS2 (v2.2.9.1). Venn diagram of (g) up-regulated (diff.log2FC > 1, *P* < 0.05) and (h) down-regulated (diff.log2FC < -1, *P* < 0.05) peaks enriched in pairwise comparisons among GF, SPF, and COV mice (n = 3 in each group). *****P* < 0.0001, ns indicates no significance, was calculated by two-sided Student’s *t*-test for two groups comparisons.


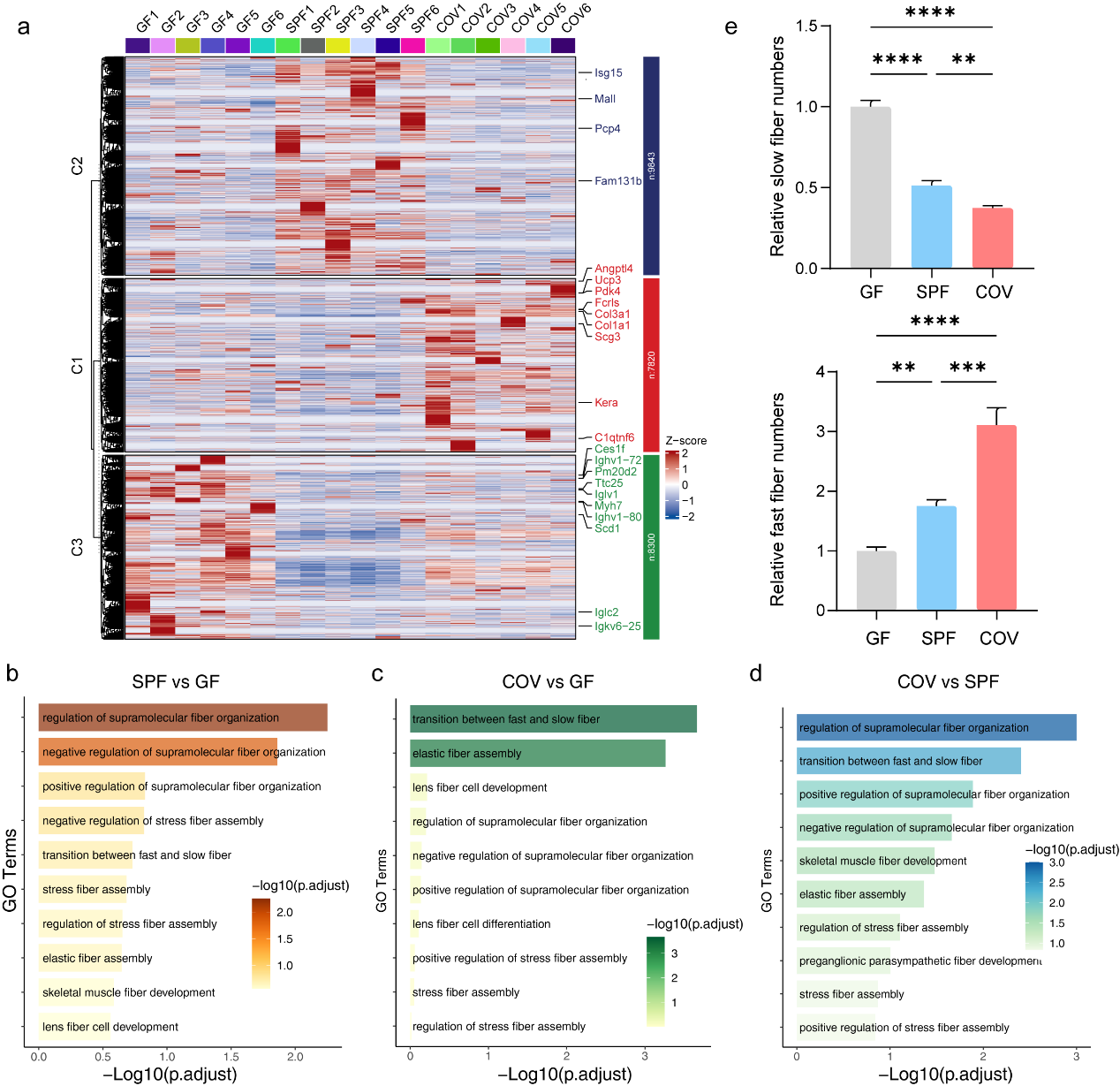


**Supplementary Figure 3.** DEGs from the GF, SPF, and COV are enriched in the fiber-related pathways. (a) Heatmap showing the expression patterns of the 23 shared DEGs identified across all three pairwise comparisons. (b, c, d) GO analysis of these gene modules revealed that DEGs were enriched in some muscle fiber developmental-related pathways in the SPF/GF (a), COV/GF (b), and COV/SPF (c), such as “regulation of supramolecular fiber organization”, and “transition between fast and slow”. (e) Quantification of slow-twitch (top) and fast-twitch (bottom) fiber types in GF, SPF, and COV mice. ***P* < 0.01, ****P* < 0.001, *****P* < 0.0001, ns indicates no significance, was calculated by two-sided Student’s *t*-test for two groups comparisons and by one-way ANOVA for three groups comparisons.


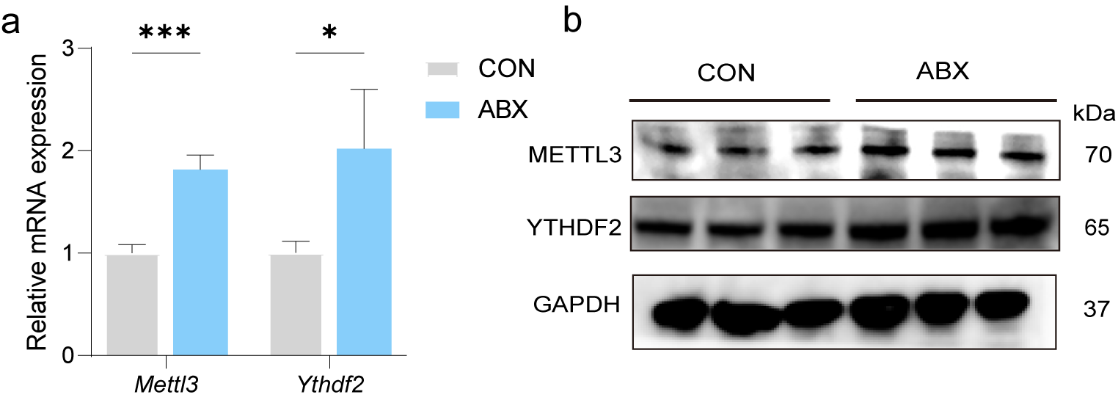
**Supplementary Figure 4.** *Mettl3* and *Ythdf2* expression in GAS muscles of ABX-treated and control mice. (a) RT-qPCR and (b) Western blot analysis of METTL3 and YTHDF2 protein expression from the CON and ABX mice (n = 3/group). Data are presented as the mean ± SEM. **P* < 0.05, ****P* < 0.001, which was calculated by two-sided Student’s *t*-test for two groups comparisons.


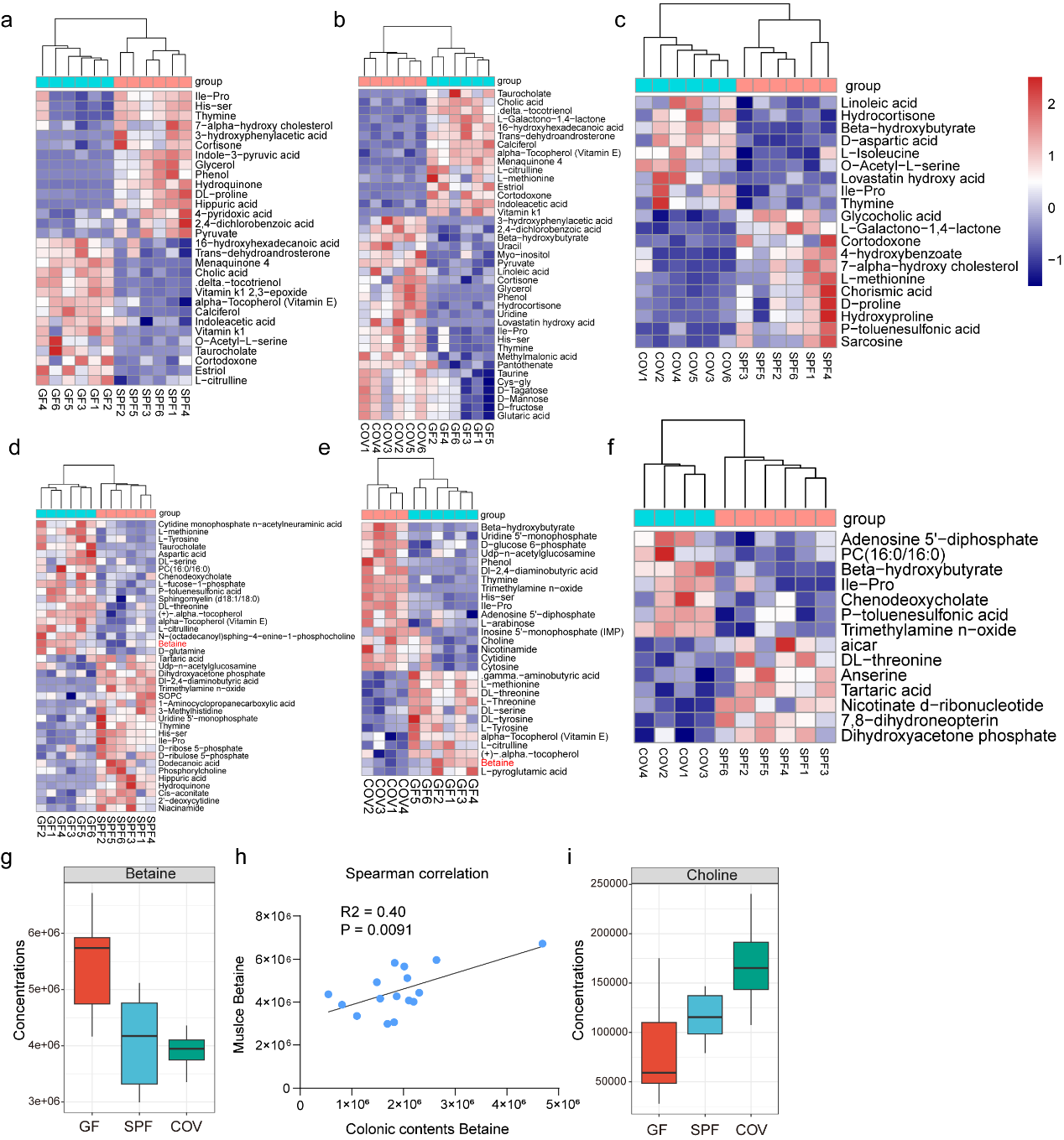
**Supplementary Figure 5.** Betaine derived from gut microbiota may regulate muscle fiber remodeling. (a-c) Heatmaps displaying the differentially expressed metabolites (DEMs) enriched in the “Metabolic pathways” of serum, comparing GF vs. SPF mice (a), GF vs. COV mice (b), and SPF vs. COV mice (c). (d-f) Heatmaps displaying DEMs enriched in the “Metabolic pathways” of skeletal muscle, comparing the same groups. (g) Box plots showing significantly different betaine concentrations in the skeletal muscle among GF, SPF, and COV mice, in which displays median (center line), 75th (upper limit of box) and 25th percentiles (lower limit of box) and outliers (whiskers) if values do not exceed 1.5 × interquartile range. (h) The correlations of betaine were positive between colon contents and skeletal muscle by Spearman (n = 6, 6, and 4 in GF, SPF, and COV mice, respectively). (i) Box plots show that the concentration of choline was different in the skeletal muscle among GF, SPF, and COV mice.


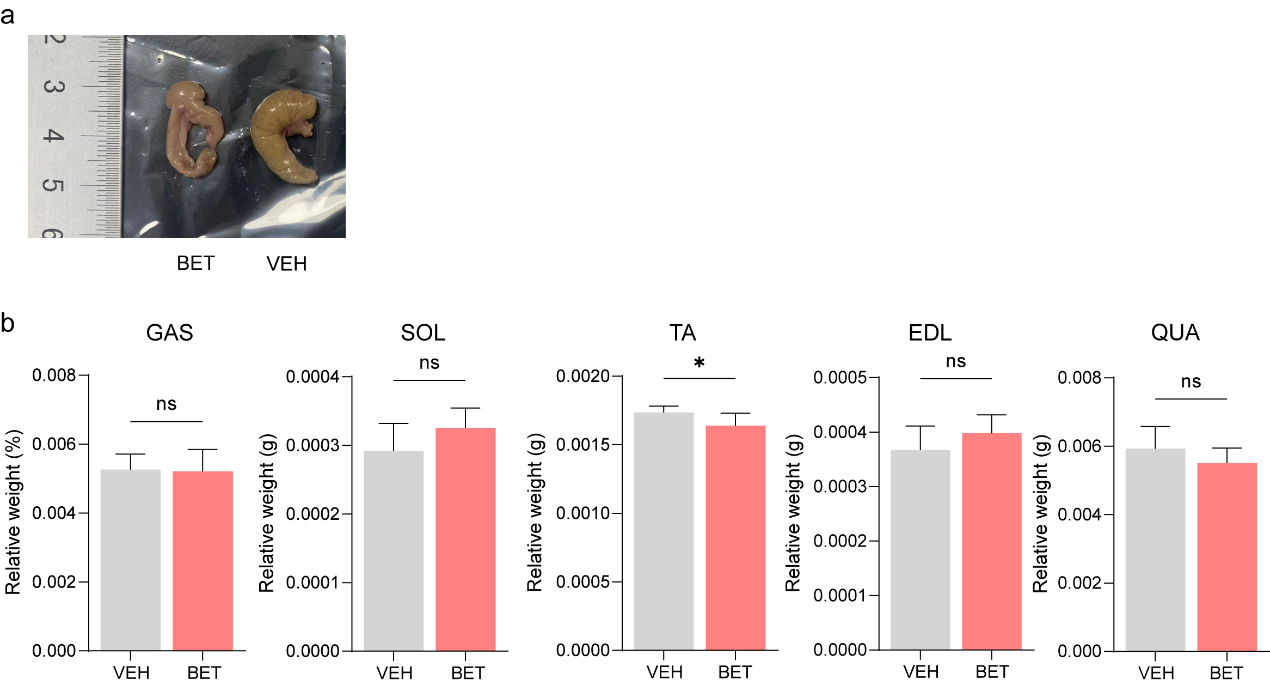


**Supplementary Figure 6.** Phenotypes characteristics of betaine-treated mice. (a) Representative images showing the cecum morphology in VEH and BET groups. (b) Weights of the GAS, soleus (SOL), tibialis anterior (TA), extensor digitorum longus (EDL), and quadriceps muscles (QUA) from VEH, and BET mice (n = 10/group). Data are presented as the mean ± SEM. **P* < 0.05, ns indicates no significance, was calculated by two-sided Student’s *t*-test for two groups comparisons.

**
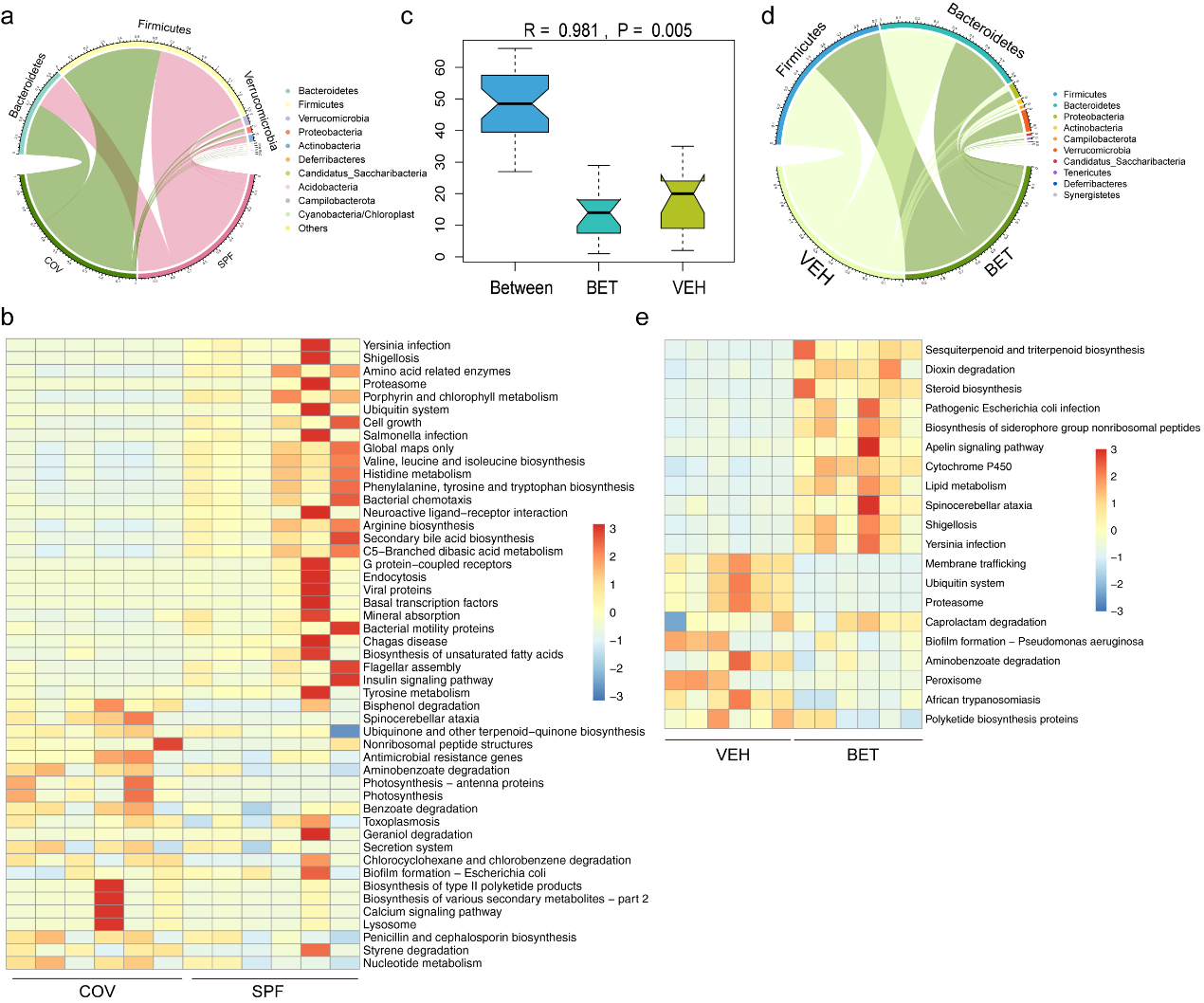
**

**Supplementary Figure 7.** Distinct gut microbiota composition across experimental groups. (a) Relative abundance of the top 10 bacterial phyla in colonic contents between SPF and COV groups. (b) PICRUSt2 analysis of the predicted functional profiles of microbial communities between SPF and COV mice. (c) ANOSIM (R = 0.98, *P* = 0.005) reveals significantly distinct microbial community structures between VEH and BET groups. (d) Relative abundance of the top 10 bacterial phyla in colonic contents between the VEH and BET groups. (e) PICRUSt2 analysis of the predicted functional profiles of microbial communities between BET and VEH mice. Differential abundance analysis was performed using the limma (v3.64.1) package with empirical Bayes moderation.
